# The Effect of Assessments on Student Motivation for Learning and Its Outcomes in Health Professions Education: A Review and Realist Synthesis

**DOI:** 10.1097/ACM.0000000000005263

**Published:** 2023-08-25

**Authors:** Rashmi A. Kusurkar, Cesar Orsini, Sunia Somra, Anthony R. Artino, Hester E.M. Daelmans, Linda J. Schoonmade, Cees van der Vleuten

**Affiliations:** 1 **R.A. Kusurkar** is professor and research programme leader, Research in Education, Amsterdam University Medical Centers location Vrije Universiteit Amsterdam, professor and research programme leader, LEARN! Research Institute for Learning and Education, Faculty of Psychology and Education, VU University Amsterdam, and professor and research programme leader, Amsterdam Public Health, Quality of Care, Amsterdam, the Netherlands; ORCID: http://orcid.org/0000-0002-9382-0379.; 2 **C. Orsini** is associate professor in medical education, Norwich Medical School, University of East Anglia, Norwich, United Kingdom, and Researcher in Health Professions Education, Faculty of Dentistry, Universidad de Los Andes, Santiago, Chile; ORCID: http://orcid.org/0000-0002-5226-3625.; 3 **S. Somra** was research assistant, Research in Education, Amsterdam University Medical Centers location Vrije Universiteit Amsterdam, Amsterdam, the Netherlands, at the time of this study.; 4 **A.R. Artino Jr** is professor and associate dean for evaluation and educational research, School of Medicine & Health Sciences, George Washington University, Washington, DC; ORCID: http://orcid.org/0000-0003-2661-7853.; 5 **H.E.M. Daelmans** is director of the master of medicine programme, Faculty of Medicine Vrije Universiteit Amsterdam, Amsterdam, the Netherlands.; 6 **L.J. Schoonmade** is information specialist at the medical library, Vrije Universiteit Amsterdam, Amsterdam, the Netherlands; ORCID: https://orcid.org/0000-0002-2407-5977.; 7 **C. van der Vleuten** is professor, School of Health Professions Education, University of Maastricht, Maastricht, the Netherlands; ORCID: http://orcid.org/0000-0001-6802-3119.

## Abstract

**Purpose:**

In health professions education (HPE), the effect of assessments on student motivation for learning and its consequences have been largely neglected. This is problematic because assessments can hamper motivation and psychological well-being. The research questions guiding this review were: How do assessments affect student motivation for learning in HPE? What outcomes does this lead to in which contexts?

**Method:**

In October 2020, the authors searched PubMed, Embase, APA PsycInfo, ERIC, CINAHL, and Web of Science Core Collection for “assessments” AND “motivation” AND “health professions education/students.” Empirical papers or literature reviews investigating the effect of assessments on student motivation for learning in HPE using quantitative, qualitative, or mixed methods from January 1, 2010, to October 29, 2020, were included. The authors chose the realist synthesis method for data analysis to study the intended and unintended consequences of this complex topic. Assessments were identified as stimulating autonomous or controlled motivation using sensitizing concepts from self-determination theory and data on context–mechanism–outcome were extracted.

**Results:**

Twenty-four of 15,291 articles were ultimately included. Assessments stimulating controlled motivation seemed to have negative outcomes. An example of an assessment that stimulates controlled motivation is one that focuses on factual knowledge (context), which encourages studying only for the assessment (mechanism) and results in surface learning (outcome). Assessments stimulating autonomous motivation seemed to have positive outcomes. An example of an assessment that stimulates autonomous motivation is one that is fun (context), which through active learning (mechanism) leads to higher effort and better connection with the material (outcome).

**Conclusions:**

These findings indicate that students strategically learned what was expected to appear in assessments at the expense of what was needed in practice. Therefore, health professions educators should rethink their assessment philosophy and practices and introduce assessments that are relevant to professional practice and stimulate genuine interest in the content.

In higher education, in general, and in health professions education (HPE), more specifically, authors have debated the effect of assessments on learning, with phrases like “assessment drives learning,” “assessment for/of learning,” and “assessment as learning” pervading the literature.^1–3^ However, the effect of assessments on the quality of student motivation for learning and its consequences have been largely neglected in this scientific dialogue. This gap is important as high-stakes assessments can not only hamper students’ autonomous motivation in the long term^4^ but also produce psychological distress.^5^ Thus, high-stakes assessment's effect on motivation could be one causal mechanism by which assessment influences learning and psychological well-being.^6,7^ In this review, we aim to improve health professions educators’ understanding of how assessments influence student motivation for learning, which in turn has an effect on learning and psychological well-being outcomes.

For this review, psychological well-being includes feeling good and functioning effectively.^7^ Thus, negative psychological well-being would be characterized as either or both of these being compromised.^7^ Learning denotes “an enduring change in behavior or the capacity to behave in a given fashion, which results from practice or other forms of experience.”^8^

Along with providing summative evaluations of students’ knowledge and skills, educators often intend for assessments to produce learning. Students, on the contrary, often focus on “giving a performance” rather than on learning.^9,10^ This represents a major gap between the intention and impact of assessments. The “2018 consensus framework for good assessment” recommends 7 criteria for assessments, including that they have educational and catalytic effects that are concerned with student motivation.^11^ Educational effects refer to assessments motivating students to prepare for and produce educational benefit, whereas catalytic effects refer to assessments providing results and feedback that motivate stakeholders in creating, improving, and supporting education.^11^ In this framework, the concept of motivation is rather limited, as it focuses on the educational benefit, while ignoring the aspect of motivation suggesting education should inspire and stimulate student curiosity.^12,13^ Lineberry puts forward the concept of “assessment affecting learning,” which considers student motivation and recommends using assessment as the primary way of encouraging learning and performance in education.^9^ But, this approach does not differentiate between driving student learning through controlled and autonomous motivation or address the problem of how driving controlled motivation has a harmful effect on autonomous motivation.^12,13^ Self-determination theory (SDT) of motivation—which classifies motivation as autonomous (out of genuine interest and/or personally endorsed importance) and controlled (out of internal or external pressure or contingent on rewards or sanctions)—can provide guidance on how assessments should be conceptualized to foster autonomous rather than controlled motivation.^12,13^ Autonomous motivation as compared with controlled motivation is associated with deep learning, better academic performance, higher creativity, and psychological well-being.^12–14^ Stimulating autonomous motivation is contingent on the satisfaction of 3 basic psychological needs: autonomy (sense of choice in learning), competence (sense of capability for learning), and relatedness (sense of belonging to the peer group). In contrast, controlled motivation is stimulated by the frustration of these needs.^12–14^

SDT posits that high-stakes assessments have deleterious effects on students’ autonomous motivation for learning and can corrupt educational practices.^4^ Even after educators distinguish between formative and summative assessments in HPE, students often perceive formative moments as summative ones. They try to control their grades by choosing their best performance moments for their formative assessments.^15^ This may be rooted in the notion that assessments often help to determine future educational opportunities. This can, therefore, happen even in sophisticated assessment systems or programs, like programmatic assessment, which is primarily designed as an assessment for learning opportunity.^16^ In medical schools that have adopted programmatic assessment, students have been found to treat formative assessments like summative assessments when they do not feel a sense of control over the assessment outcome.^17^ This is especially true for knowledge-based assessments with structured answers, when there is a lack of a trusting relationship with teachers and when assessments cannot be used for improving performance.^17^ Moreover, teachers may implement assessments differently than intended by curriculum developers (e.g., different clinical supervisors have been found to apply standards for scoring competencies differently).^3,18^ This is an additional factor that can widen the gap between the intention and impact of assessments. Thus, even a well-intentioned assessment system or program may work against student motivation if implemented incorrectly.^19^

Therefore, this review aims to examine the effect of assessments on motivation and its consequences, as reported in the HPE literature, thereby attempting to provide a scientific grounding for designing assessments that stimulate student curiosity and autonomous motivation which, in turn, should ultimately foster learning and long-term clinical performance and psychological well-being. The research questions guiding this review were: How do assessments affect student motivation for learning in HPE? What outcomes does this lead to in which contexts?

## Method

We conducted our search in a systematic manner and used realist synthesis method for data analysis. We used the cited realist reviews published in HPE along with methodology articles to guide our method.^20–24^

### Search strategy

The search strategy was developed iteratively with an information specialist (L.J.S.) and was limited to 2010–2020 because of feasibility considerations.^21^ In October 2020, a comprehensive search was performed in 6 bibliographic databases—PubMed, Embase, APA PsycInfo, ERIC (Education Resources Information Center), CINAHL, and Web of Science Core Collection—that included articles from January 1, 2010, to October 29, 2020, with no language restrictions. The following terms were used in 3 iteratively developed search strings (including synonyms and closely related words) of index terms or free-text words: “assessments” AND “motivation” AND “health professions education/students” (see Supplemental Digital Appendix 1 at http://links.lww.com/ACADMED/B420 for the full search strategy). Duplicate articles were excluded. A snowball search was conducted on the references of all included articles to identify more relevant articles.

### Inclusion criteria

Articles were included if they investigated the effect of assessments (all types) on student motivation for learning in HPE; were empirical papers and literature reviews; and used quantitative, qualitative, or mixed methods.

### Exclusion criteria

Articles were excluded if they examined a non-HPE population, were not on assessments and motivation, were nonempirical publications (opinions, perspectives, letters, editorials, commentaries, dissertations, conference abstracts), or if motivation was not an outcome measure or was not measured or evaluated.

### Rigor and relevance

The rigor and relevance of included articles were evaluated as specified by the RAMSES (Realist and Meta-narrative Evidence Syntheses: Evolving Standards) standards of publication for a realist synthesis.^20,21^ Rigor evaluated the credibility and trustworthiness of the method used to generate the results. Relevance evaluated the importance of the article in answering our research questions.^20^

### Rationale for choosing realist synthesis

We wanted to study the intended and unintended consequences of a complex topic—the effect of assessments on motivation and hence on, among others, learning and psychological well-being outcomes. Because realist synthesis explores “which mechanisms lead to what outcomes in which contexts” (context–mechanism–outcome), it seemed to be the most suitable method for our analysis.^20,21^ That said, we did not follow the realist review method described by Carrieri and colleagues because our intention was not to find an immediately practically applicable intervention (e.g., to determine which intervention is best for tackling doctors’ and medical students’ mental ill-health by involving important stakeholders at all stages).^25^ Our intention was instead to study the context–mechanism–outcome configurations of assessment features (e.g., assessment content, format) that influence motivation and hence produce, among others, learning and psychological well-being outcomes. Thus, the realist method followed by other authors, who also studied factors influencing certain variables in HPE, suited our objectives better.^21–24^

### Data extraction

R.A.K. and C.O. or S.S. first read all titles and abstracts to make inclusion or exclusion decisions followed by reading full texts to make further inclusion or exclusion decisions. Differences of opinion were discussed in a meeting and resolved through consensus. R.A.K. and C.O. or S.S. then independently extracted actual data sentences or phrases from the articles (see Supplemental Digital Appendix 2 at http://links.lww.com/ACADMED/B420), which was finalized through consensus. R.A.K then extracted context–mechanism–outcome data (see Supplemental Digital Appendix 3 at http://links.lww.com/ACADMED/B420), which was independently checked by C.O. and S.S. Differences in opinion were resolved through consensus.

### Data analysis

R.A.K. conducted all steps of the analysis. C.O. and S.S. checked each step independently. A.R.A. Jr checked the coded data of 6 randomly chosen articles.

First, R.A.K. used MAXQDA (version 2020, VERBI GmbH, Berlin, Germany) to conduct a content analysis of the data using sensitizing concepts from SDT to identify stimulation of autonomous or controlled motivation.

Second, she classified the codes from the content analysis as a context, mechanism, or outcome. The conditions which led to effects on autonomous or controlled motivation constituted the context. How autonomous or controlled motivation was stimulated constituted the mechanism. Outcomes comprised learning and psychological well-being outcomes, among others (e.g., decreased inspiration, creation of a performance culture).

Third, R.A.K. extracted context–mechanism–outcome configurations to propose overarching program theories on how assessment features stimulate autonomous or controlled motivation. The realist program theories proposed here were finalized through consensus among the whole research team.

## Results

**Figure 1:**
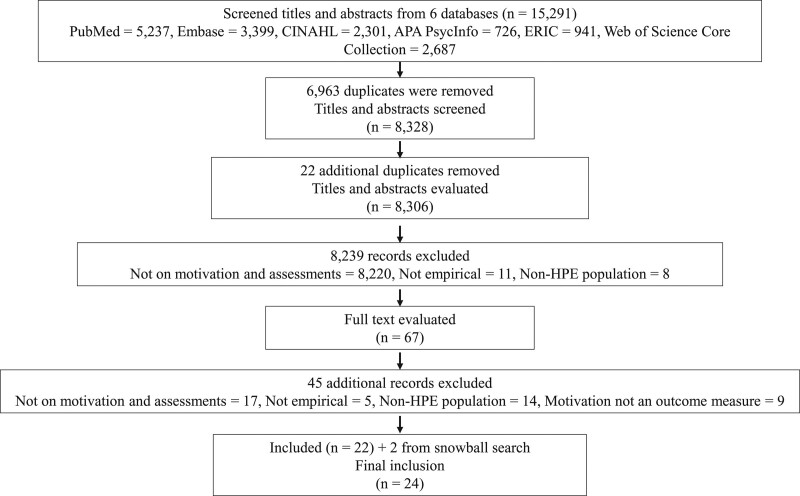
Flow diagram of the review process for an October 2020 review and realist synthesis aiming to examine the effect of assessment on motivation and, among other, learning and psychological well-being outcomes, as reported in the HPE literature. Abbreviation: HPE, health professions education.

After applying the inclusion and exclusion criteria, 24 out of 15,291 articles were included (see Figure 1).^26–49^ The rigor and relevance of each article is reported in Supplemental Digital Appendix 2 (at http://links.lww.com/ACADMED/B420).

Ten studies were conducted on medical students or residents; 4 on nursing students; 1 on medical and nursing students; 2 on nutrition students; and 1 each on veterinary medicine, physiotherapy, health sciences, oral health, physician assistant, pharmacy, and dental students. Eleven studies were conducted in Europe, 4 in Australia, 3 each in Asia and the United States, 2 in Canada, and 1 in the Middle East. As we used a realist synthesis approach, we focused on the features of the different assessments in this review. The specific assessments were, therefore, not relevant, but are listed in Supplemental Digital Appendix 4 (at http://links.lww.com/ACADMED/B420).

**Chart 1:**
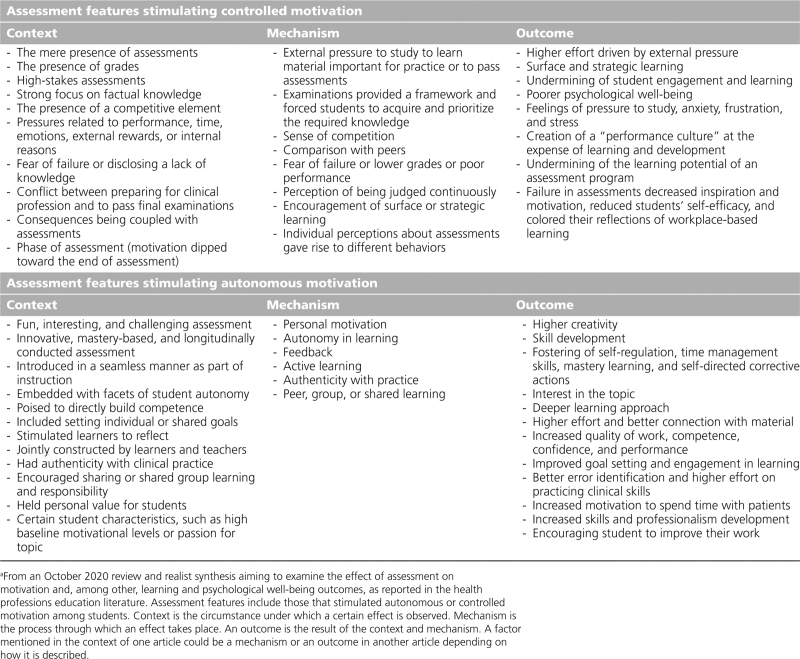
Overview of the Main Findings on Context-Mechanism-Outcome Configurations of Assessment Features That Stimulate Autonomous or Controlled Motivation^a^

In our analysis, we used sensitizing concepts from SDT, to identify which assessment features stimulated autonomous versus controlled motivation. We report the contexts, mechanisms, and outcomes for each of these 2 broad categories below (see Chart 1 for an overview of the main findings).

### Controlled motivation

#### Outcomes.

The outcomes of assessments stimulating controlled motivation included higher effort driven by external pressure^26–30^; surface and strategic learning (learning only what is likely to be assessed, which happened at the expense of learning out of interest or for future practice)^26–28,31–34^; undermining of student engagement and learning^34^; feelings of pressure to study, anxiety, frustration, and stress^26,27,33^; creation of a “performance culture” at the expense of learning and development^33^; and undermining of the learning potential of an assessment program.^33^ With assessments that stimulate controlled motivation failure in assessments decreased inspiration and motivation,^35^ reduced students’ sense of self-efficacy, and colored their reflections of workplace-based learning.^33^ Pressure to study, anxiety, frustration, stress, decreased inspiration, and low perceived self-efficacy can all be considered indicators of negative psychological well-being.^7^

#### Contexts.

Stimulation of controlled motivation happened in different contexts. The mere presence of assessments (formative^31,32^ or summative^26–28,36,37^) was an external motivator for learning or preparing for the assessments, as were the presence of grades,^34,38^ high-stakes assessments (like licensure examinations^36^),^26^ and a strong focus on factual knowledge.^27^ Further contexts stimulating controlled motivation included the presence of a competitive element^26,31^; pressures related to performance,^27^ time,^26–28,35^ emotions,^27^ external rewards,^27^ and internal reasons (the desire to fulfill supervisor’s expectations)^36^; and fear of disclosing a lack of knowledge or failure.^26,27^ Conflict between preparing for the clinical profession and to pass the final examinations^26,38^ and consequences being coupled with assessments also stimulated controlled motivation.^39^ The phase of the assessment seemed to play a role as motivation seemed to dip toward the end of the assessment.^40^

#### Mechanisms.

Assessments stimulated controlled motivation through several mechanisms. External pressure exerted to study to learn material that was important for practice^28^ or to study to pass assessments stimulated controlled motivation.^27,31^ Examinations provided a framework and forced students to acquire and prioritize the required knowledge.^28^ External pressures and rewards functioned as triggers for controlled motivation.^27,29,31,38,41,42^ Assessments triggered controlled motivation, which made students study and practice for assessments.^27^ Some formative assessments stimulated students’ controlled motivation and hence achievement.^31^ Grading of assessments motivated students to submit high-quality work.^43^ Questions asked in a formative assessment garnered more study effort if they were perceived to be important for final examinations.^31^ Although assessments made students feel pressure to study more, they could also stimulate an interest in the subject.^27^ The existence of external regulating bodies and regulations stimulated lifelong learning.^36^ Focus on entrustment as the assessment outcome seemed to enhance learner perceptions of being judged continuously and of assessments being perceived as summative rather than formative.^33^ This effect can undermine the learning potential of an assessment program, which is based on trust and psychological safety.^33^ Because of its focus on autonomy and unsupervised practice, entrustment language can lead to a performance culture at the expense of learning and development.^33^ Assessments also led to anxiety and frustration.^26,27,40,41^

Assessments also stimulated controlled motivation through creating a sense of competition or out of fear of failure or poor performance. Peer assessment led to comparisons with peers,^42^ fear of receiving low marks (if they did not contribute),^29^ and motivated them to learn. Wanting to know what their peers thought of their work prompted them to reread their work in light of the peer feedback.^42^ Students studied harder for the reward of extra credits^27^ and out of fear of getting lower grades, failing,^26,29^ or performing poorly.^30^ Competition motivated students to prepare for assessments.^26,31^ Students studied hard for collaborative tests to avoid disappointing fellow students; this strengthened their confidence and made them excel.^44^

Assessments stimulated controlled motivation resulting in encouragement of surface and strategic learning. Focus on factual knowledge in assessments and controlled motivation led to surface learning.^27^ Students who learned by rote memorization and regurgitated the material without understanding it during examinations tended to fare better on certain assessments.^26^ When traditional grading was employed, students valued the assessment on the basis of the weight it carried for the overall grade. This influenced their engagement with the topic and the learning objectives.^34^ Choosing what to study was based on previous examination questions rather than on the knowledge essential for practice.^28^ When possible, students were inclined to pick easier assessment activities to fare better.^26^ High-stakes assessments encouraged a surface learning approach, while other assessment types encouraged a deep learning approach owing to the lower stakes.^26^ Making certain assessment types (like entrustable professional activities [EPAs]) high stakes may give rise to strategic learning behavior among students.^33^

Individual perceptions about assessments gave rise to different behaviors (e.g., some students perceived generating ideas, in an active learning-based-assessment, as a competitive process, while others perceived it as an opportunity for reflection^40^).

### Autonomous motivation

#### Outcomes.

The outcomes of assessments that stimulated autonomous motivation were higher creativity^34^; skill development^44^; fostering of self-regulation, time management skills, mastery learning, and self-directed corrective actions^30,34,41^; interest in the topic^27^; a deeper learning approach,^26,43,45,46^ higher effort and better connection with the material^34,47^; increased quality of work,^29^ competence, confidence, and performance^26,40^; improved goal setting and engagement in learning^33,44^; better error identification and higher effort on practicing clinical skills^48^; increased motivation to spend more time with patients^38^; increased skills and professionalism development^45^; and encouraging students to improve their work.^42^ Lower effort was found in formative compared with summative assessments in spite of formative assessments stimulating autonomous motivation.^38^ Reflection on assessments enhanced student motivation, learning, and well-being.^33^

#### Contexts.

Stimulation of autonomous motivation happened in the context of the assessment being fun, interesting, and challenging^26,27,36,40,45^; innovative, mastery-based, and conducted longitudinally^33,34^; introduced in a seamless manner as part of instruction^46^; and embedded with facets of student autonomy (e.g., students graded themselves on mastery criteria predetermined by the teacher,^34^ use of pass/fail grading only,^34^ provision of multiple attempts to pass,^26^ choices of assignments,^34^ choices in learning about topics of interest to them,^40,45^ choices in place and time of assessment^39^).

Stimulation of autonomous motivation also happened if the assessment was poised to directly build competence and was an embedded active learning assessment, with timely, external, and multiple sources of feedback.^28,31,33,40,41,44,47,49^ Assessments that included setting individual^40^ or shared goals,^41^ stimulated learners to reflect,^33^ were jointly constructed by learners and teachers,^45^ had authenticity with clinical practice,^26,28,35,37,38,49^ encouraged sharing or shared group learning and responsibility,^27,39,41,44^ and held personal value for students also stimulated autonomous motivation.^27,31,45^ Certain student characteristics also form the context for autonomous motivation stimulation, such as high baseline autonomous motivational (for learning) levels,^39^ having the motivation for personal achievement or satisfaction, or having a love of learning and passion for the topic.^26,40^ Variations in the effect of assessments on motivation were seen depending on the type of assessment (i.e., testing fact recall or deep thinking).^26^

#### Mechanisms.

Assessments stimulated autonomous motivation through several mechanisms. A fun and challenging assessment triggered autonomous motivation and doing it in a group provided a holistic picture, which would be difficult for individual students to do on their own.^27^ Students were more driven by personal motivation when they could choose their own topics and change their learning approach.^45^ Students worked to a schedule because they were autonomously motivated.^32^ Some students would study for the assessment even if they were not graded as they just wanted to be good doctors.^38^ Formative assessments motivated students by making them aware of what they already knew and what they needed to study.^27^ Certain assessment types stimulated autonomous motivation by providing instant feedback through rubrics and shifting the focus to mastery learning.^34^

Autonomy in learning also stimulated autonomous motivation. Use of portfolio made students more personally motivated and so they did not rely only on lecture material for their study.^45^ Some amount of choice in assessments (e.g., of topics to study or what to add in the portfolio) removed boundaries (e.g., being confined to the curriculum), allowed for personal exploration, and increased students’ appreciation of the study topics.^45^ Students’ motivation increased as they progressed through the planning process and became more independent.^45^ Autonomy in learning made students independently develop their skills and increased their motivation.^40^ Being able to watch their own performance motivated students to practice their skills.^48^ Certain assessment types motivated students to study more, focus on important concepts, and reflect on their learning.^31^ Some assessment types increased motivation by giving immediate feedback.^41^ Knowing and understanding what was expected in the assessment helped students to improve.^42^

Getting feedback stimulated autonomous motivation in several ways. Face-to-face feedback improved students’ competence and confidence.^26^ Feedback from patients and colleagues motivated students to improve their competence and pursue excellence.^49^ Getting to know the correct answer immediately after the assessment stimulated students to focus more on all questions and their motivation.^31^ Assessment followed by explanatory feedback enhanced the learning process and sustained student motivation.^46^ Error detection helped students to identify their knowledge gaps.^47^ Collaborative testing helped in closing a performance gap through constant peer evaluation and feedback.^44^ By providing a better picture on their development, reflection on assessments improved students’ motivation, learning, and well-being.^33^

Active learning assessments stimulated autonomous motivation. They led to increased skills, confidence, and motivation after the completion of each stage of the learning journey.^40,47^ Active learning assessments not only supported knowledge building but also engaged students cognitively and emotionally,^42,45^ as they generated enquiry by providing students with the opportunity to formulate questions.^45^

Assessments that had authenticity with practice motivated students intrinsically as they provided a sense of wholeness, fostered clinical skills and professionalism, captured students’ interest, and encouraged teamwork.^27,38,41,44,45^ Having choices in what to include in the portfolio allowed students to include their personal experiences into their study and significantly motivated them.^45^ The professional responsibility of physicians motivated students for lifelong learning.^36^

Peer, group, or shared learning helped students to identify their knowledge gaps, created constructive friction, and moved them into Vygotsky’s Zone of Proximal Development (i.e., the zone in which students have enough challenge and are motivated to learn new things), enhancing their learning.^41^ Peer assessment functioned as an extra motivational strategy for individual students to contribute to the group in a meaningful way and provided teamwork experience.^29,44^ Peer learning formats led to interactions, resulting in engagement and motivation.^44^ The peer assessment process empowered students, motivated them, and increased their confidence and engagement in learning.^42^ Group assessment helped students to get a broader view of topics, which would have been hard to achieve on their own.^27^ Learning in a group with a shared interdependent goal made students feel personal responsibility for the group work and contribute more in terms of knowledge and effort.^41^ Some students perceived generating ideas as part of the assessment as a competitive process, while others perceived it as an opportunity to reflect on their ideas and make comparisons.^40^ Failure led students to study harder.^33^ Not passing their entrustment assessment made students feel frustrated.^33^

### Emergent program theories based on context–mechanism–outcome combinations

**Figure 2:**
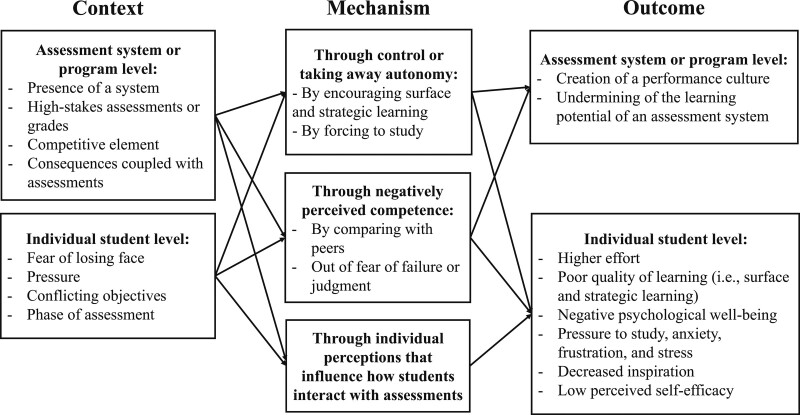
Realist program theory schematic showing how assessments can enhance controlled motivation and lead to negative learning and psychological well-being outcomes, from an October 2020 review and realist synthesis aiming to examine the effect of assessment on motivation and, among other, learning and psychological well-being outcomes, as reported in the health professions education literature.

Figure 2 depicts the emergent realist program theory that explains how assessments can enhance controlled motivation and lead to negative learning and psychological well-being outcomes. Negative psychological well-being was an outcome reported only in qualitative data and was characterized by negative emotions, such as anxiety, stress, and frustration.^26,27,33^ For an understanding on how assessments can enhance controlled motivation, we found contextual factors at an assessment system or program level as well as at an individual student level that work by taking away autonomy and creating negative perceptions of competence. We also found that at an assessment system or program level, stimulation of controlled motivation led to creation of a performance culture and undermining of the learning potential of the assessment system.

**Figure 3:**
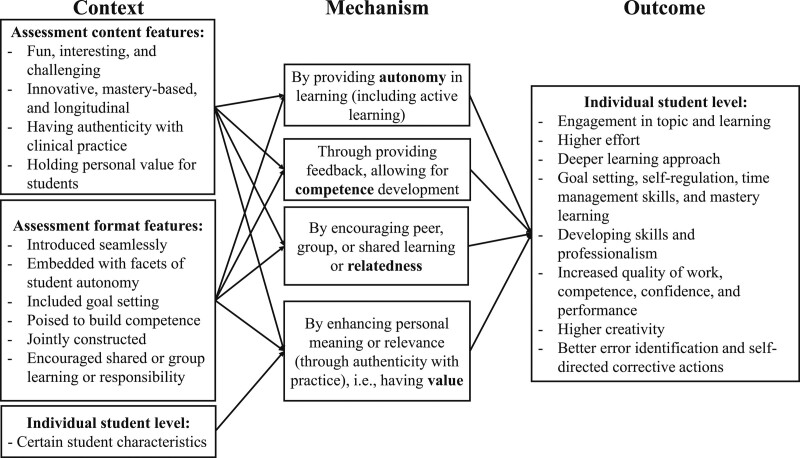
Realist program theory schematic showing how assessments can enhance autonomous motivation and lead to positive learning outcomes, from an October 2020 review and realist synthesis aiming to examine the effect of assessment on motivation and learning and, among other, psychological well-being outcomes, as reported in the health professions education literature. No psychological well-being outcomes were found for assessments that stimulate autonomous motivation.

Figure 3 depicts the emergent realist program theory that explains how assessments can enhance autonomous motivation and lead to positive learning outcomes. For insights into how assessments can enhance autonomous motivation, we found that contextual factors in the assessment features (i.e., in the assessment content and format, as well as at an individual student level) work through satisfying the basic psychological needs of autonomy, competence, and relatedness as well as by creating value for the activity to produce positive learning outcomes at an individual student level. We did not find psychological well-being outcomes for assessments that stimulate autonomous motivation.

## Discussion

In this review and realist synthesis, we sought to add to the HPE literature by describing context–mechanism–outcome configurations for how assessments influence motivation leading to learning and psychological well-being outcomes among students at an individual level as well as at an assessment system or program level. We found that assessments, at the assessment system or program as well as the individual student levels, enhance controlled motivation by frustrating the SDT-based psychological needs of autonomy and competence, leading to negative outcomes at the assessment system or program as well as the individual student levels. In contrast, we found that certain assessment as well as individual student characteristics enhance autonomous motivation through satisfaction of the SDT-based basic psychological needs of autonomy, competence, and relatedness, along with creation of value to produce outcomes only at an individual student level. We did not find any psychological well-being or assessment system- or program-level outcomes related to the enhancement of autonomous motivation in the current HPE literature.

**Chart 2:**
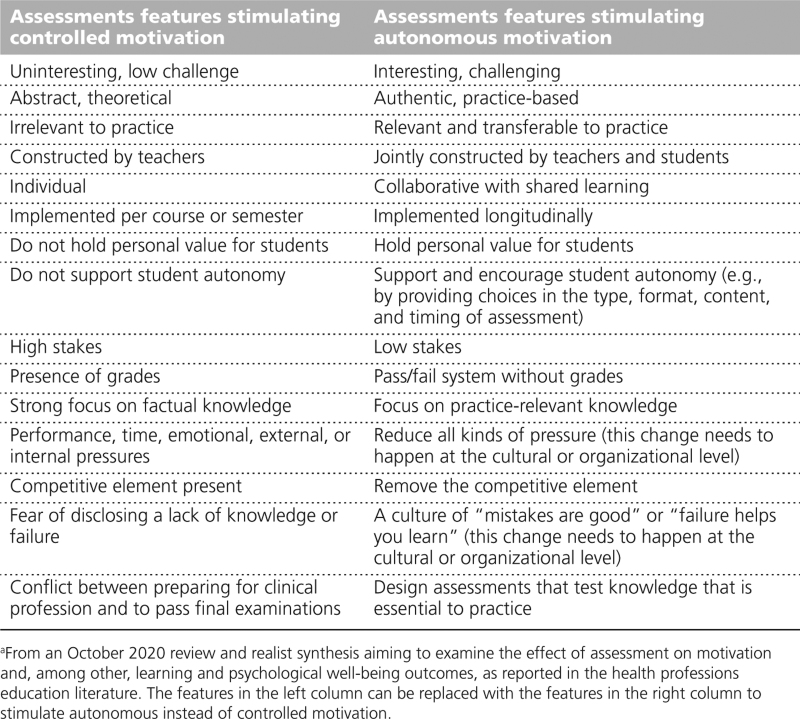
How Educators Can Convert Assessments That Stimulate Controlled Motivation Into Ones That Stimulate Autonomous Motivation by Making Changes to the Assessment Features^a^

Based on our findings, in Chart 2, we provide a list of feature changes educators can use to convert assessments that stimulate controlled motivation into ones that can stimulate autonomous motivation. This is important because assessments that stimulate controlled motivation can not only produce negative psychological well-being outcomes but also have a long-term deleterious effect on autonomous motivation for learning.^4^

In current HPE practice, the 2 major assessment concepts being implemented widely are programmatic assessment and EPAs.^50^ We did not find any research on the effect of programmatic assessment on student motivation. Such research would add to the literature, especially because programmatic assessment has some of the features identified in this review as ones that seem to stimulate autonomous motivation.^51^ In terms of EPAs, we found one study suggesting that EPA-based assessments mainly stimulated controlled motivation.^33^ This finding was somewhat surprising, given that EPA-based assessments have some of the features identified in this review as stimulating autonomous motivation (e.g., authenticity with clinical practice combined with providing students with autonomy).^52^ We hypothesize that this disconnect may be due to the gap between the design of individual EPA assessments and how they are embedded in an assessment program or an overall assessment culture, but this needs further investigation.

The presence of a gap between the intention and impact of an assessment, which was seen throughout the results of this review, aligns with earlier published literature.^3^ Tensions between different assessment practices, the implementers’ beliefs about assessments, and the requirement to uphold and guarantee the quality of graduating trainees may account for this gap.^53^ This issue can be addressed by a multipronged strategy that includes considering student motivation when designing assessments, changing the culture within which assessments are embedded, training faculty to implement assessments as they are intended,^3^ and building a shared understanding between teachers and students on the goals of assessment.^54^

### Implications for practice

The assessment features we found that can stimulate autonomous motivation can be used by educators to redesign current assessments or develop innovative assessments. We recommend developing assessments that more authentically prepare students for clinical practice, such as having more clinical reasoning exercises, where students describe patient problems and the underlying mechanisms, or diagnostic justification exercises, where students suggest a differential and rationale, over the standard multiple-choice questions. We found a trend that suggests students strategically learned what was expected to appear in the assessments at the expense of what was needed in practice. This is alarming in light of SDT’s claim that stimulation of controlled motivation through incentives (i.e., grades and qualifications) has a long-term deleterious effect on autonomous motivation.^4,55^ Only if educators pay attention to stimulating autonomous motivation for learning through assessments, will they be able to “light the fire of learning” instead of just “filling the bucket” for HPE students.^13^

### Implications for further research

The results of this review provide a scientific basis for developing a research program on designing innovative assessments stimulating autonomous motivation and investigating how they work through design-based research and the effects of programmatic and EPA-based assessments on student motivation.

### Limitations

This review was limited to the HPE literature. A broader review of the effect of assessments on motivation in higher education may be beneficial. Although we evaluated the rigor and relevance of each study in the review, we did not use this evaluation to exclude articles. This approach, however, fits well with the realist synthesis method,^20,24^ allowing us to include all ideas that contribute to innovative assessment methods.

## Conclusions

Assessment features stimulating controlled motivation seemed to lead to negative consequences like decreased psychological well-being. Assessment features stimulating autonomous motivation seemed to lead to positive outcomes such as higher effort and creativity. Our findings indicate that students strategically learn what is expected to appear in assessments at the expense of what is needed in practice. This approach leads to stress and negative psychological well-being. Therefore, health professions educators urgently need to rethink their assessment philosophy and practices and introduce assessments that stimulate curiosity and genuine interest in the content and that are relevant to professional practice.

## Acknowledgments:

The authors would like to thank Joyce Kors, MSc, Anouk Wouters, PhD, Malou Stoffels, MSc, Jettie Vreugdenhil, MSc, and Lianne Mulder, MA, MPhil, from Amsterdam University Medical Centers, Faculty of Medicine Vrije Universiteit Amsterdam, and Andries Koster, PhD, from University of Utrecht, for their feedback on an earlier version of this article.

## Supplementary Material
