# Supplementary material for: The Effect of Assessments on Student Motivation for Learning and Its Outcomes in Health Professions Education: A Review and Realist Synthesis
Source: Acad Med. 2023 Aug 25;98(9):1083–92. doi: 10.1097/ACM.0000000000005263 (PMC10453393; doi:10.1097/ACM.0000000000005263)
Supplement: Supplementary file 1 [file acm-98-1083-s001.pdf]

Supplemental digital content for Kusurkar RA, Orsini C, Somra S, et al. The effect of assessments on student motivation for learning and its outcomes in health professions education: A review and realist synthesis. Acad Med.

Supplemental Digital Appendices 1–4

Table of Contents

Supplemental Digital Appendix 1: Full Search Strategy……………………………………………………………………………2

Supplemental Digital Appendix 2: Data Extraction Table of Included Studies …………………………………………………………8

Supplemental Digital Appendix 3: Context-Mechanism-Outcome Data Extracted From the Included Studies………………………...41

Supplemental Digital Appendix 4 : Type of Assessments Used in the Included Studies………………………………………………..50

Supplemental Digital Appendix 1
Full Search Strategy

Research Question:
How do assessments affect student motivation for learning in HPE? What outcomes does this lead to in which contexts?

Researcher: Rashmi Kusurkar
Information Specialist: Linda Schoonmade
Date: October 29, 2020

Results October 29, 2020

Databases	Results
PubMed	5237
Embase.com	3399
Cinahl (Ebsco)	2301
PsycInfo (Ebsco)	726
Eric (Ebsco)	941
Web of Science	2687
**Total**	**15291**
**After Deduplication**	**8328**

Search Histories October 29, 2020

PubMed History and Search Details October 29th 2020 (5237)

Search	Query	Results
#5	#1 AND #2 AND #3 Filters applied: from 2010/1/1 - 3000/12/12	5,237
#4	#1 AND #2 AND #3	8,264
#3	"Educational Measurement"[Mesh] OR Assessment[tiab] OR exam[tiab] OR exams[tiab] OR examinati*[tiab] OR educational measurement*[tiab]	1,889,356

Supplemental digital content for Kusurkar RA, Orsini C, Somra S, et al. The effect of assessments on student motivation for learning and its outcomes in health professions education: A review and realist synthesis. Acad Med.

Search	Query	Results
#2	"Motivation"[Mesh:NoExp] OR "Goals"[Mesh] OR motivat*[tiab] OR goal*[tiab]	516,813
#1	"Students, Health Occupations"[Mesh] OR "Clinical Clerkship"[Mesh] OR Student*[tiab] OR intern[tiab] OR internship[tiab] OR resident[tiab] OR residents[tiab] OR registrar[tiab] OR postgraduate[tiab] OR clerk[tiab] OR undergraduate*[tiab]	484,489

Embase.com History and Search Details October 29th 2020 (3399)

Search	Query	Results
#6	#5 NOT ('chapter'/it OR 'conference abstract'/it OR 'conference review'/it OR 'editorial'/it OR 'erratum'/it OR 'letter'/it OR 'note'/it OR 'short survey'/it)	3,399
#5	#1 AND #2 AND #3 AND [2010-2020]/py	6,386
#4	#1 AND #2 AND #3	8,479
#3	'assessment'/exp OR (assessment OR exam OR exams OR examinati* OR “educational measurement*”):ab,ti,kw	2,529,520
#2	'motivation'/exp OR (motivat* OR goal*):ab,ti,kw	689,010
#1	'health student'/de OR 'medical student'/exp OR 'paramedical student'/exp OR 'public health student'/exp OR 'veterinary student'/exp OR 'clinical education'/exp OR (student* OR intern OR internship OR resident OR residents OR registrar OR postgraduate OR clerk OR undergraduate*):ab,ti,kw	652,161

Eric (Ebsco) History and Search Details October 29th 2020 (941)

Search	Query	Results
S6	S5 AND Filter applied: Academic Journals	941
S5	S1 AND S2 AND S33 AND [2010-2020]/py	1,194
S4	S1 AND S2 AND S3	1,948
S3	'assessment'/exp OR (assessment OR exam OR exams OR examinati* OR “educational measurement*”):ab,ti,kw	199,749
S2	'motivation'/exp OR (motivat* OR goal*):ab,ti,kw	184,544
S1	DE ("Students" OR "Nursing Students" OR "Undergraduate Students" OR "Medical Students" OR "Graduate Students") OR TI ((student* OR intern OR internship OR resident OR residents OR registrar OR postgraduate OR clerk OR undergraduate*) AND (medical OR medicine OR dental* OR nursing* OR pharmacy* OR "public health" OR "health occupation*" OR midwifery OR clinical OR veterinary)) OR AB ((student* OR intern OR internship OR resident OR residents OR registrar OR postgraduate OR clerk OR undergraduate*) AND (medical OR medicine OR dental* OR nursing* OR pharmacy* OR "public health" OR "health occupation*" OR midwifery OR clinical OR veterinary)) OR KW ((student* OR intern OR internship OR resident OR residents OR registrar OR postgraduate OR clerk OR undergraduate*) AND (medical OR medicine OR dental* OR nursing* OR pharmacy* OR "public health" OR "health occupations" OR midwifery OR clinical OR veterinary))	81,229

Cinahl (Ebsco) History and Search Details October 29th 2020 (2301)

Search	Query	Results
S6	S5 AND Filter applied: Academic Journals	2,301
S5	S1 AND S2 AND S3 AND [2010-2020]/py	2,453

Search	Query	Results
S4	S1 AND S2 AND S3	3,719
S3	MH ("Educational Measurement" OR "Achievement Tests" OR "Credentialing Examinations+" OR "Computerized Educational Testing+" OR "Clinical Competence+" OR "Test Taking" OR "Competency Assessment" OR "Professional Competence") OR TI (assessment OR exam OR exams OR examinati* OR educational measurement*) OR AB (assessment OR exam OR exams OR examinati* OR educational measurement*)	198,874
S2	MH ("Motivation" OR "Achievement" OR "Academic Achievement" OR "Commitment" OR "Excellence" OR "Intention") OR TI (motivat* OR goal*) OR AB (motivat* OR goal*)	550,127
S1	MH ("Students, Health Occupations" OR "Students, Allied Health" OR "Students, Midwifery" OR "Students, Medical" OR "Students, Dental" OR "Students, Nursing+" OR "Students, Nursing, Practical" OR "Students, Pharmacy" OR "Students, Audiology" OR "Students, Dental Hygiene" OR "Students, Dietetics" OR "Students, Medical Technology" OR "Students, Occupational Therapy" OR "Students, Physical Therapy" OR "Students, Physician Assistant" OR "Students, Respiratory Therapy" OR "Students, Radiologic Technology" OR "Students, Social Work" OR "Students, Speech-Language Pathology" OR "Students, Graduate+" OR "Students, Nursing, Graduate+" OR "Students, Undergraduate") OR (TI (student* OR intern OR internship OR resident OR residents OR registrar OR postgraduate OR clerk OR undergraduate*) AND (medical OR medicine OR dental* OR nursing* OR pharmacy* OR "public health" OR "health occupation*" OR midwifery OR clinical OR veterinary)) OR (AB (student* OR intern OR internship OR resident OR residents OR registrar OR postgraduate OR clerk OR undergraduate*) AND (medical OR medicine OR dental* OR nursing* OR pharmacy* OR "public health" OR "health occupation*" OR midwifery OR clinical OR veterinary))	173,773

APA PsycInfo (Ebsco) History and Search Details October 29th 2020 (726)

Search	Query	Results
S6	S5 AND Limiters: Academic Journals	726

Search	Query	Results
S5	S1 AND S2 AND S3 **Limiters** - Publication Year: 2010-2021	1,062
S4	S1 AND S2 AND S3	1,884
S3	DE ("Performance Tests" OR "Educational Measurement" OR "Curriculum Based Assessment" OR "Entrance Examinations" OR "Formative Assessment" OR "Grading (Educational)" OR "Minimum Competency Tests" OR "Reading Measures" OR "Curriculum Based Assessment" OR "Formative Assessment" OR "Grading (Educational)" OR "Minimum Competency Tests OR "Professional Examinations") OR TI (assessment OR exam OR exams OR examinati* OR educational measurement*) OR AB (assessment OR exam OR exams OR examinati* OR educational measurement*) OR KW (assessment OR exam OR exams OR examinati* OR educational measurement*)	498,211
S2	("Achievement Motivation" OR "Motivation" OR "Academic Achievement Motivation" OR "Goal Orientation" OR "Goals" OR "Educational Aspirations" OR "Goal Setting" OR "Approach Avoidance" OR "Aspirations" OR "Aspiration Level" " OR "Extrinsic Motivation" OR "Incentives" OR "Educational Incentives" OR "Intention" OR "Behavioral Intention" OR "Intrinsic Motivation") OR TI (motivat* OR goal*) OR AB (motivat* OR goal*) OR KW (motivat* OR goal*)	426,128
S1	DE ("Medical Students" OR "Nursing Students" OR "Dental Students" OR "Graduate Students" OR "Postgraduate Students" OR "Undergraduate Students") OR TI ((student* OR intern OR internship OR resident OR residents OR registrar OR postgraduate OR clerk OR undergraduate*) AND (medical OR medicine OR dental* OR nursing* OR pharmacy* OR "public health" OR "health occupation*" OR midwifery OR clinical OR veterinary)) OR AB ((student* OR intern OR internship OR resident OR residents OR registrar OR postgraduate OR clerk OR undergraduate*) AND (medical OR medicine OR dental* OR nursing* OR pharmacy* OR "public health" OR "health occupation*" OR midwifery OR clinical OR veterinary)) OR KW ((student* OR intern OR internship OR resident OR residents OR registrar OR postgraduate OR clerk OR	86,487

Search	Query	Results
	undergraduate*) AND (medical OR medicine OR dental* OR nursing* OR pharmacy* OR "public health" OR "health occupation*" OR midwifery OR clinical OR veterinary))	

Web of Science Core Collection History and Search Details October 29th 2020 (2687)

Search	Query	Results
#5	#4 Timespan=2010-2020	2,687
#4	#1 AND #2 AND #3	3,673
#3	TS= (assessment OR exam OR exams OR examinati* OR educational measurement*)	913,257
#2	TS= (motivat* OR goal*)	2,303,251
#1	TS= ((student* OR intern OR internship OR resident OR residents OR registrar OR postgraduate OR clerk OR undergraduate*) AND (medical OR medicine OR dental* OR nursing* OR pharmacy* OR "public health" OR "health occupation*" OR midwifery OR clinical OR veterinary))	213,709

Google Scholar Search String

Motivation|goal+education+exam|goal|assessment+student|intern|clerk|resident|registrar|postgraduate|undergraduate+medicine|dental|nursing|pharmacy|health|midwifery|clinical|verinary

Supplemental Digital Appendix 2

Data Extraction Table of Included Studies

Reference	Aim, Design & Method	Setting	Participants	Results - Mechanism and outcome	Perspective	Level of contribution
Ahmad 2018[32]	Aim: To investigate the learning styles and preferences of medical students and thus guide medical teachers to improve the quality of teaching and learning.<br>Design: Cross-sectional.<br>Method: Mixed – Quantitative questionnaire followed by interviews.	Pakistan	1st and 3rd year medical students (n=90 for quantitative part and n=12 for qualitative part) in surgical rotation	"Besides, 18(33%) and 14(39%) students of third and final years studied due to some motivation or stimulus of pre-test or examination."<br>"Some learners are strategic and they are affected by the assessment system and plan to get through the tests and exams and achieve high scores."<br>"The same is evident in response to item no. 01 of our study where 18(33%) and 14(39%) students of third and final years admitted that their study was driven by in-house tests and exams."<br>"Extrinsic motivation plays an important role in study habits of medical students".<br>"In item one of our study, nearly 40% of students in both the groups worked according to a schedule due to their intrinsic motivation".<br>"This situation reflects the need and importance of motivation provided by in-house formative assessment system".<br>"The formative assessment system is a very vital drive for students because all of them admitted that they studied on motivation of in-house tests".<br>"Formative assessment system is an important motivation for students. Their learning preferences, superficial, deep or strategic, are mostly determined by the assessment system adopted in in-house tests and examinations."	Medical students	Low<br>Rigor - -<br>Relevance +
Alkhamees	Aim: To evaluate the study habits of Saudi urology	Saudi Arabia	Registered urology	"The most influential factor motivating seniors to study was the final board exam (P = 0.006), while that for juniors was	Urology residents	Low<br>Rigor - -<br>Relevance +

2020[37]	residents throughout their residency training.<br>Design: Cross-sectional<br>Method: Quantitative questionnaire-based		residents from years 1-5 (n=142)	preparation for the operating room (OR)". "For both groups, the least motivating factor was preparation for their clinics".<br>"The final board exam was found to be the highest motivating factor affecting urology residents' study time."<br>"The second most motivating factors for both groups in our study were preparation for the OR and the annual end-of-year exam".<br>"Lai et al reported that exams, site-specific patient cases, and presentations are top motivators for study".		
Asghar 2010[41]	Aim: The purpose of the study reported here was to qualitatively explore whether the supposed benefits of RPC as a formative assessment strategy are achieved.<br>Design:<br>Method: Qualitative phenomenological approach	UK	1st year Physiotherapy students (n=12)	"Used as a formative assessment strategy, Reciprocal Peer Coaching (RPC) has the capacity to increase motivation in students due to the nature of the shared interdependent goal, and to provide immediate feedback to students on completion of the assessment."<br>"Cognitive development theory suggests that it can create critical cognitive conflicts between peers, and that the discomfort this produces motivates students to explore gaps in knowledge and understanding (Ladyshewsky 2001; Slavin 1996)."<br>"The interaction between peers allows students to enter the zone of proximal development where a less able peer is able to enter a new area of potential development through problem-solving with someone more able (Vygotsky 1978)."<br>"Motivating learning - Three key elements were considered as motivators to learn by the first-year students, time pressures, emotional pressures and feedback or the desire to understand where a student was in relation to their goals."<br>"Most of the first-year students interviewed felt that RPC formative assessment opportunities were a good thing, motivated them to learn and helped them to manage time."<br>"The pressure of imminent assessment prompted increased time being spent on task during RPC practice, in comparison	Physiotherapy students	Very Low Rigor – Relevance +/-

				to during ordinary lesson time, when students admitted messing about."<br>"It is possible that the 'pressure' of having to perform in forthcoming real life clinical practice also encouraged meaning to be embedded in memory, rather than just remembering the words to pass the test."<br>"Whereas in class, you just … your concentration might wander a little bit. You don't concentrate maybe as much, but if you're made to do something, like in the assessment, then you know that it's important and you do try and pay attention the best you can." "Formative assessment in any form may play an important role in improving students'<br>time management skills and assisting them to develop self-regulatory processes and it is arguable that RPC acted as an incentive to ensure that a student's time was used constructively. Students repeatedly talked about the pressure they experienced and it seemed that this pressure focused attention and prompted them to use their time more effectively."<br>"For some students, their most immediate motivation was to impress others, to receive the extrinsic reward of positive feedback, that pat on the back for all to see."<br>"Responsibility as an individual for your own learning has its own set of emotional baggage; however, the emotional aspects of shared learning and assessment brought up a number of different issues: shared empathy for failure and shared enjoyment of success; a sense of potential individual humiliation in front of others in your group in the face of failure, as well as the shame of letting your group down; frustration from those students who felt it would be much better if they alone were responsible for their own success or failure, who felt disgruntled with the demands of participating in group work."		

				"Learning in a group towards a mutually interdependent goal prompted feelings of personal responsibility for the rest of the team, which helped to motivate students to learn, bring knowledge, improve their skills and practice together."<br>"Vicarious reinforcement was reported in the study with students modifying their behavior as a consequence of their observations of each other, but it should not necessarily be viewed negatively as this can provide rich opportunities for feedback and promotes learning (Ladyshewsky 2001)."<br>"This suggests that there is a need to structure activities to build self-efficacy and that formative assessment, such as RPC through setting short-term goals to act as cognitive motivators and through the feedback it provides, helps students believe in themselves and their capabilities (Bandura 1997)."<br>"Pintrich (2000) suggests three types of goals: performance goals, where individuals are motivated to strive to out-do others; mastery goals, where personal achievement is what matters and performance avoidance goals, where individuals are negatively motivated to avoid looking incompetent."<br>"Appraisal of where someone might see themselves in relation to their goal, and how individuals judge the personal meaning of the situation, influences the range of emotions to emerge and whether those emotions will sustain or reduce motivation (Pekrun et al. 2002; Schutz and DeCuir 2002)."<br>"		
Bengtsson 2010[35]	Aim: The aim of this study was to gather information regarding what students consider important for their motivation to attain	Sweden	16 nursing students from years 1 and 3 and 15	"Medical students learn in general more for life than the nursing students, who instead had their focus on passing their assessment".<br>"It's the time that creates the limitation of how and how much to learn. I need to pass the exam. Otherwise it will be difficult to catch up again"	Nursing and medical students	Low<br>Rigor -<br>Relevance +

	knowledge, and consider how we can use that information to shape courses that foster their motivation to learn.<br>Design: Interview study.<br>Method: Qualitative.		medical students from years 1 and 6.	"if the nursing students failed, for example on a test and had to catch up, their inspiration and motivation decreased."<br>"Their primary goal was to learn for life, whereas the nursing students had their assessments in focus since they expired that they had no time to read all that they wanted."<br>"Therefore, it is important to point out that assignments and examinations should reward deeper knowledge, and a test should not only be an end-of-the course exercise to determine the students' grades".<br>"A test should be followed up in a behavioristic perspective in which the knowledge (Biggs, 2003) and the skills (Miller, 1990), should be set in conjunction with each other and focus on the students profession".		
Casey et al. 2011[42]	Aim: This paper describes how Peer Assessment was implemented in an undergraduate nursing program as a method of enhancing student engagement, and reports on students' experiences of the process.<br>Design: Focus group study.<br>Method: Qualitative descriptive Interpretative.	Ireland	Bachelor of Nursing students (n=37)	"There is also a consensus that Peer Assessment (PA) motivates student learning, as it increases their interest in the topic, engages them in the assessment process, and encourages them to present themselves positively to their peers."<br>"They appeared to be more knowledgeable, reporting a better understanding of what was expected in their assignments. This prompted students to think more deeply about how they approached the assignment and motivated them to learn and improve their work".<br>"Students were eager to learn what their peers thought of their work. This prompted them to reread their own assignment in light of their peers' feedback, which they did rarely with the lecturer's feedback".<br>"The PA process thus empowered students, motivated them to learn, and increased their confidence, ultimately enhancing their engagement in learning". (Results)<br>"The PA learning activity motivated students to learn, as they read their feedback eagerly and evaluated their performance in relation to their peers. Unfortunately, the same eagerness and	Nursing students	High Rigor ++ Relevance ++

				level of engagement did not apply when traditional assessment methods were used”.<br>“The literature also revealed that PA motivated students to focus on how they could improve their work in the future”.		
Cobb et al. 2013[26]	Aim: The aim of this study was to evaluate the impact of two different assessment formats (DOPS – Direct Observation Procedural Skill, and MCQ – Multiple Choice Questions) on the approaches to learning (including learning motive and strategy) of final year veterinary students.<br>Design: Survey + Interview.<br>Method: Mixed method, quantitative questionnaire-based (SPQ)+Thematic analysis of qualitative interview data.	UK	Veterinary Final Year students (n=87)	The scores of Surface, deep and achieving motives have not been reported separately, but rather as a combined (motive + strategy=approach) score. We therefore do not report the quantitative results in this review.<br><br>“DOPS encouraged the search for deeper understanding”.<br>“I think at the moment it (MCQ examination) sort of biases towards people who can absorb facts, absorb facts, absorb facts, and then spew it out for a week of assessment, rather than sort of testing the more rounded sort of characteristics of an individual and a sort of deeper understanding of the material.” (Context?)<br>“I would just go and cram for it and just try and get any exposure to that skill until I did the DOPS. It was sheer panic. I can’t describe how scared we were that we weren’t going to get them done. That’s the only thing we thought about. On a Monday morning when you started rotations, am I going to get a DOPS, am I going to get to do it. That really drove us. But then having said that, the last two or three rotations, cos we’d finished, we got them all done by end of March, we actually really relaxed and we had more time to sit and learn about the cases we’d seen and chat about the cases.” (Context?)<br>“I always start my revision as I should mean to go on, which is sort of going through things in-depth and trying to understand them. Inevitably I run out of time and have to resort to flicking through lectures and skim-reading things.” (Context?)	Veterinary students	High Rigor ++ Relevance ++

				"Obviously I'm going to do that before an exam cos I know it might get me the marks, but I don't feel happy that that's the way I'm learning."(Outcome?)<br>"I'll be honest, I did tactically pick certain DOPS so they could only fall on certain rotations. And I tactically picked the easier DOPS out of different skill areas, cos that's just sensible."(Mechanism?)<br>"Cos I'm interested in small animals, whereas with equine and farm, sometimes I might not be quite aware of all the important things. So I'd ask other people about it, just sort of discuss with each other what the important diseases were, and really focus on those and make sure I have a good understanding of those, and then everything else comes as sort of a bonus." (Mechanism?)<br>"So you tore yourself away from something interesting to go get a urine sample cos you wanted to practise cos you really wanted to pass. Yet there's going to be a hundred chances to get a urine sample, but they might be doing something really interesting over there. It was a bit of a hard dilemma cos you felt like it shouldn't be the focus, but yet at the back of your mind you think I've got to pass this so I need to practise it." (Mechanism?)<br>"Almost all the students in this study demonstrated a deep motivation to learn, often expressed in terms of wanting to become a 'good vet' and do the best they can for their clients and to ensure the welfare of animals in their care." (Mechanism?)<br>"It gives you feedback about your own performance and your own understanding, knowledge and whereabouts you are, especially whereabouts you are in the year. I think that's quite important cos we're quite a competitive year." (Mechanism?)		

				"Some also described the competitive element associated with the final examination."<br>"Achieving motivation extended beyond wanting to become a 'good vet'. For some students motivation to learn comes from their own personal gain and the satisfaction of high attainment." (Mechanism?)<br>"Some described deep, intrinsic motivation to learn: wanting to learn for their own satisfaction and a 'love of learning'."<br>"Some students reported that 'fear of failure', a surface motivator had a strong influence."(Mechanism?)<br>"My revision leave was purely and simply so I did not fail cos I was so scared of failing and not graduating with my mates." (Outcome?)<br>"I think they (DOPS) are generally a good way of assessment. I think it does make you think about what you need to know and certainly you sort of get used to saying whether you're competent or not and then that kind of transfers to other skills and you sort of think well can I do this, could I do it on day one." (Mechanism?)<br>"the participants considered that high stakes assessment of MCQs encouraged a surface approach whereas the lower stakes assessment of DOPS prompted a deeper, more reflective approach." (Context?)<br>"If you know a vet's going to quiz you, you'll spend much more time looking stuff up. If you know they're not going to ask questions, inherently human nature's not to look so much stuff up, and it probably shouldn't be the way, but invariably it is."(Context?)<br>"I'm quite okay at practical skills, but OSCEs, you just get so stressed and your hands are shaking, I don't think it's a very realistic way of kind of assessing practical skills really. I think that DOPS do that a lot better because it's in a real setting, you know, probably the best way of doing it. An MCQ I don't		

				think particularly represents what we're going to do when we're out there in practice, because you don't have an option of four things to choose from." (Context?)<br>"Participants appreciated the regular opportunity for face to face feedback in WPBA. It helped them to improve and boosted their confidence." (Outcome?)<br>"In contrast to the MCQ examination, time pressure for DOPS was less of an issue for most students."(Context?)<br>"I think with the DOPS, if you fail one, you have the time to, you know, pass 2 more in the group and get the group done, you know, and think about it build on it and reflect on okay, why did I fail. And that's, in my opinion, very good, because it gives you time to use that experience and build on it."(Context?)<br>"Both DOPS and MCQ assessment formats impacted on student learning. However, these effects were not uniform across the participants." (Mechanism?)<br>"The learning outcomes assessed may be as important to the approach adopted as the<br>assessment format itself. Evidence from the qualitative findings of this study indicates that the effects may be due as much to the stakes involved as to the format of the assessment."(Mechanism?)<br>"High stakes assessment, such as final examinations, can be a powerful driver for learning but the impact is not necessarily a positive one for all students (Cilliers et al. 2010; Al-Kadri et al.2012). Low stakes assessment are likely to lead to deeper approaches to learning (Nicol & Macfarlane-Dick 2006; Al-Kadri et al. 2012). Students will employ surface-learning strategies when under time pressure or stressed, at the expense of deeper more meaningful learning which they know will be beneficial to their future career, assessment therefore has the potential to inhibit learning for clinical practice. But a few		

				will use deep learning strategies even when these are not conducive to achievement. However for this sample of students, who were nearing graduation, the achieving approach was relatively constant regardless of the assessment format."(Mechanism?)<br>"Salient in the students' experience is the conflict between preparing for their clinical profession and preparing to pass final examinations. The resolution of these conflicts is a challenge for assessors as well as for students."(Context?)		
Elliott 2005[29]	Aim: The purpose of this study was to develop a self and peer assessment strategy which would promote student participation and satisfaction with group<br>Work and evaluate it.<br>Design: Survey study.<br>Method: Mixed; Quantitative (Survey) and qualitative (open comments in the survey).	Ireland	17 postgraduate nursing students	"Another important finding to emerge from this study was that self and peer assessment had a positive impact on student motivation."<br>"Sixty-five percent of students (n =11) believed that the self and peer assessment increased their own motivation with 53% (n=9) agreeing that it increased their colleague's motivation to participate in the group work."<br>"The theme of motivation was also common in the students' qualitative comments."<br>"Student stated that the self and Peer assessment was an 'extra motivational strategy to keep all the group going' and 'it provides motivation for group member to make a meaningful contribution to the group work' and furthermore 'it encouraged a higher standard of work and promoted attendance at meetings'. However, for one student fear was a motivating factor as the following comment suggests; 'it definitely made me work as there was the fear of being marked down if I didn't contribute'."	Nursing students	Low Rigor - Relevance +/-
González-Gil	Aim: To determine the effectiveness of 360-degree evaluation for	Spain	67 third year nursing students	"I think what motivates me the most about this project is getting feedback from patients and colleagues, because I think that's what's really important. When we are no longer students and have to work, we have to know what our strengths are	Nursing students	Low Rigor – Relevance +/-

2020[49]	assessing the competencies acquired by third-year nursing students during their clinical placements.<br>Design:<br>Method: Mixed method (quantitative assessment data + qualitative focus group data)		(quantitative data) 10 students + 4 teachers (2 focus group for qualitative data)	and, if we have never thought about it, if we haven't even given it a thought about how we're doing with respect to our team and with respect to the patients, well, then we won't know what to do there and we won't have any experience on how to improve."<br>"Overall, and despite requiring a significant effort by all of the participants in the evaluation, the 360-degree evaluation proposal was perceived to be complete and comprehensive, representing a motivating initiative for change and a commitment to the pursuit of excellence."<br>"Students and teaching staff (CIs) have rated the experience as highly satisfactory, describing it as comprehensive, integrated, motivating, and learning-oriented."		
Harris 2011[43]	Aim: To determine the reliability and effectiveness of peer assessment in a large enrollment class.<br>Design: Longitudinal mixed methods study.<br>Method: Quantitative assessment data + qualitative open-ended survey-based written feedback.	UK	Approx. 180 Year-1 BSc Physiology students	""A number of students reported that preparing for the peer assessment session had increased their motivation in completing the report: "It increased the effort I put into completing the pro forma" and "I usually forget about practicals once I've done them but this makes sure that I follow them up and actually understand them.""<br>"Free text feedback from the participating students also showed that the summative aspect of the mark awarded provided strong motivation to submit high-quality work, with frequent comments such as "Made me work harder thinking it was going to get marked."<br>"Since the renal practical report contributed only 2% to the overall course mark, the discrepancies incurred were considered to be tolerable, particularly in view of the very high correlation between staff and peer marks and the motivational benefits (confirmed by student feedback) of the small summative component attached to the mark awarded."	Physiology students	Medium Rigor - Relevance ++

				"Although students find peer assessment challenging, their feedback shows that it can encourage them to "think more," "learn more," and "reflect more on their work." (Mechanism?)		
Ismail 2019[31]	Aim: To determine the benefits of using Kahoot! for formative assessment in education.<br>Design: Cross-sectional.<br>Method: Qualitative focus group based.	Malaysia	36 pre-clinical medical students	'When we play Kahoot!, we feel like we are playing something. We like it because we are in a stressful environment for the entire day, but when we have Kahoot! sessions, we see it as a game, sort of entertainment.' At the same time, we get knowledge.' 'For me, the Kahoot! session is helpful because of its competitiveness. Psychologically, when there is a competition, we will get ready for it. So, it will encourage us to continuously learn.'<br>'I used to think that, okay, this question is not that important, so just go through. I don't go in deep. So, when they ask during Kahoot!, I'll think … oh, this is important. There is a chance for them to ask this kind of question. So, I go back to my room and study.'<br>'Kahoot! triggers us to cover the topics properly.'<br>'We cover everything so that we can answer Kahoot! properly. So, this automatically improves our academic performance.'<br>'When we play it together, it's really fun. When we play it, we know our ranking. If we notice our ranking is at the bottom … [we will think] after this: I will focus … must answer the questions correctly. With the usual assessment, we do not know [immediately whether our answer is correct] … One more thing: At the end of the session, there are prizes for the winners. So, this will motivate us more….'<br>'Kahoot! always encourages us by telling us "You are almost there…." … "Try harder…." It's motivating us….'<br>'When you fail something, it will trigger you to work more and to get it right….'	Medical students	High Rigor ++ Relevance ++

				‘When our names are displayed [on the Kahoot!ers of the month frame], we feel very proud … It is worth what we have [gone through to] learn.’<br>“Kahoot! sessions increased their motivation through competition, which increased their self-confidence and stimulated their participation in more sessions.”<br>‘… and another thing is Kahoot! is more challenging because the time is like … very short. We have 20 s for each question.…’<br>‘The Kahoot! session is something like a class … we just go there … attend the session … answer the questions and then we can come out happy.…’<br>‘We feel really excited … fun … because we can see [the result] live [on the screen].’<br>‘It is an attractive learning tool that is a source of motivation and guidance for student learning.’<br>‘Formative assessment can have a significant effect on student motivation and achievement if it is designed to stimulate the extrinsic aspects of student motivation.’<br>‘Its competitive, environment is key.’<br>‘Kahoot! sessions motivate students to study, focus on the important concepts and reflect on<br>what they have learned.’		
Leadbeatter 2018[45]	Aim: To investigate students’ engagement with basic science courses following introduction of the portfolio.	Australia	21 oral health students	“The results showed that the students successfully interweaved personal experience into their studies and that it provided significant motivation for learning.”<br>“The integrative and scaffolded assessment was generally regarded as both challenging and interesting. All participants spoke]about how liberation from topic selection motivated them to complete and perform well in the assignment, but also influenced their approaches to learning basic science and their wider studies.”	Oral health students	High<br>Rigor ++<br>Relevance ++

	Design: Cross-sectional.<br>Method: Qualitative, focus groups.			Interest and motivation - "Students demonstrated willingness to put more effort into the assessment and experienced more positive emotions about learning."<br>A sense of personal control – "Students took more control and responsibility over their own learning for the assessment."<br>"By giving us the freedom and choice to research things that we found interesting, I feel as if it made me appreciate research and [basic science] immensely."<br>"I was very on top of things. Because I was doing something I was interested in, if didn't have<br>anything to do, I would go on when I had some spare time."<br>"A sense of personal control over learning was highlighted by many. The consequences of giving students this choice were reflected in the way many students commented about the far-reaching effects of removing boundaries and allowing personal exploration. Some students noted that, after the assignment, the way they approached learning changed from relying on lecture material to being driven by a much more personal motivation."<br>"Participants stated that the assessment generated enquiry by providing them the opportunity<br>to formulate questions in which they were interested."<br>"Relevance, mentioned several times by participants in the focus groups, had a threefold meaning. First, the topics selected by students related to their own lives, and they could weave their own interests and experiences into their research. Second, students were engaged through topics that were not confine by the course curriculum, and they were able to relate their own topic to other subjects within and even beyond basic science. Third, the assessment was viewed as relevant to future oral health practice and patient care and, as such, contributed to the development of clinical skills and professionalism."		

				"Participants agreed that the assessment not only supported knowledge-building, but it also occupied them emotionally and cognitively." "You learn one thing, you have to do something a certain way. [But] when you are able to choose what you wanted to do, you were more motivated .." "The findings of this study suggest that a sense of personal control is a critical starting point for many of the effects that the assessment had on student learning. Along with choice, interest in the topic was another important aspect."		
Makransky 2020[46]	Aim: To determine if there was a significant increase in intrinsic motivation, self-efficacy, and transfer from pre- to post-test after using the VR genetics simulation as a classroom learning activity. Design: Pre-test post-test design. Method: Quantitative, survey-based.	Denmark	208 medical students	"Items within a simulation were found to fit the PCM and the results showed that the sample had a small significant increase in intrinsic motivation and self-efficacy, and a large significant increase in transfer following the genetics simulation." "One of the main assumptions underlying innovative assessment methods such as stealth assessment (Shute 2011) or Bayesian nets (Almond et al. 2015), is that assessment should be seamlessly introduced in educational material without interrupting students' in the learning process." "The results suggest that the MC questions with explanatory feedback enhance the learning process and sustain students' motivation." "In the genetics simulation questions were carefully developed as an integrated part of the simulation, where students learn through answering the questions. Students are rewarded based on the number of times it takes them to answer correctly; and all students are given an explanation of the correct response before moving on in the simulation, thereby ensuring that students gain fundamental knowledge of the topic. The results support the large body of literature that	Medical students	High Rigor ++ Relevance ++

				has found formative assessment and immediate feedback to improve motivation and learning.”<br>“Results showed that the sample had a small significant increase in intrinsic motivation and self-efficacy, and a large significant increase in transfer, following the genetics simulation.”		
Martin 2020[33]	Aim: To explore trainee perceptions of the impact of EPAs and entrustment scales on feedback and learning processes in the clinical setting.<br>Design: Cross-sectional.<br>Method: Qualitative (focus groups).	Canada	4 focus groups with 17 PG trainees in anesthesia, emergency medicine, general internal medicine and nephrology	“Furthermore, residents expressed concerns that EPA-based assessments might lead to strategic behaviors if assessment data were to be used in high-stakes decisions about progress and competence development. Trainees described feeling tempted to select assessment opportunities with the goal of ‘passing’ an EPA or ‘looking good’, rather than learning.”<br>“This requires careful balancing of holistic and analytic approaches in EPA design. This seems to be of particular importance to trainees when they try to use EPAs to guide their learning: if the EPAs are too broad, they provide no benefit in guiding goal setting or the selection of relevant learning experiences; however, if they are too narrow, other learning opportunities that are not captured may be ignored, trivialized or undervalued.”<br>“Trainees reported that the use of entrustment scales impacted their sense of self-efficacy and colored their reflections on workplace-based learning experiences: if residents did not achieve a sufficient entrustment score when evaluated using an EPA, they described feeling frustrated about their entire clinical experience that day.”<br>“EPAs and entrustment scales can support trainees' goal setting and learning they also carry the risk of being turned into tick-box exercises.”<br>“Some participants in this study noticed a decreased drive to continue to develop task-specific competencies once they had 'achieved competence.”	Anesthesia trainees	Medium/High<br>Rigor ++<br>Relevance +

				"The use of entrustment language, with its focus on autonomy and unsupervised practice, may in fact reinforce a 'performance culture' at the expense of a focus on continuous development and learning."<br>"Trainees clearly expressed a desire to be successful in achieving the EPA and felt significant anxiety and frustration when use of the entrustment scale did not result in a maximum score reflecting the achievement of 'competence'. Participants did, in fact, express<br>concerns about the impact of the entrustment scale, irrespective of its anchors, on their self-efficacy, motivation and stress levels."<br>"Assessment systems based on EPAs and the inherent focus on the assessment outcome (i.e., entrustment) may thus enhance learner perceptions of continuously being judged and of assessments being summative rather than formative, thus undermining the learning potential of an assessment programme based on trust and psychological safety."<br>"Stimulating learner reflection over multiple EPA-based assessments can provide a longitudinal and more meaningful view of development and strengthen learners' confidence, motivation and well-being."		
Paxman 2011[40]	Aim: To compare the motivation, confidence, and control of nutrition students undertaking an "active learning" assessment during their learning journey.	UK	65 Final Year Nutrition UG-students for survey, 2 focus groups one with 6	<u>Initial briefing</u> - "Before the initial briefing (pre1; n = 64), levels of motivation were explained, in most cases, by students feeling anxious (24)." "After the briefing (post1; n = 64), student levels of motivation were still largely explained by feelings of anxiety; however, only 14 students reported this. Others most commonly reported feeling excited (12), inspired (11), and interested (10)." "After the briefing, students reported that they felt "excited," a comment<br>that was supported by the high levels of motivation at this stage." *"The reason for the "excitement" was that the*	Nutrition students	High<br>Rigor ++<br>Relevance ++

	Design: Pre-post activity design.<br>Method: Quantitative survey with a limited number of open narrative answers, followed by focus groups with students and faculty.		students and another with 7 faculty	*students were able to take part in an autonomous, self-selected piece of work about which they felt passionate."*<br>Thought shower - "Before the thought shower (pre2; n = 65), levels of motivation were once again primarily explained by heightened anxiety (19) though some students reported feeling interested (12)." "After the thought shower (post2; n = 63), levels of motivation improved with students mostly reporting feeling inspired (17) or enthusiastic (14)." *"The thought shower produced interesting responses as some of the respondents saw the generation of ideas as a "competitive process" while others saw it as time to reflect on the ideas and make comparisons. Regardless of the language used, both answers had a strong element of competition." "the students had sought autonomy but this level of autonomy could be daunting and therefore the "safety blanket" of their tutor was thought necessary.'*<br>Business proposal presentation – "Naturally, before their oral business proposal presentation (pre3; n = 65), levels of motivation dipped because many students felt anxious (34)." "After they had delivered their oral business proposal presentations (post3; n = 65), levels of motivation improved, albeit only slightly. " *"Importantly from the point of view of autonomy, it made the students "work on their own and develop their own skills." Further, as one respondent phrased it, they "had to be professional." It was at this stage that the students developed an awareness of their developing skill sets."*<br>Feedback on proposal – "Once they had received their feedback on both the oral and written business proposal tasks (post4; n = 60), their levels of motivation were mostly explained by anxiety (19),"<br>Nutrition fair – "Before their final task, the Nutrition Fair itself (pre5; n = 55), the students were mainly motivated by		

				anxiety (28).” “After the Fair (post5; n = 50), motivation dipped perhaps because their assessment tasks had come to an end for the module; most students reporting feeling inspired (16).” *“On the day of the Fair, the students were “frantic” and felt they were “running on adrenaline.” It is interesting to note that these feelings coincided with relatively high levels of motivation.”*<br>“Similarly, females were significantly more motivated than males following the initial briefing (P = 0.002) but at no other stage.”<br>“As level of knowledge and skills improved through each stage of the learning journey, students gained confidence, which could explain the increases in motivation seen after the completion of each stage. Each stage involved activities that epitomize autonomous<br>learning and unsurprisingly, many of the students displayed characteristics of autonomous learners described by Cotterall (1995).”<br>“Throughout the journey, the students had to set themselves goals in order to efficiently complete the challenges that had been either set for them or that they had set themselves.”<br>“It is generally agreed that learners become more autonomous the more motivated they are (Deci and Ryan 1985; Dickinson 1995; Dornyei and Csizer 1998).”<br>“As the students progressed through the planning process, and consequently became<br>more independent, levels of motivation increased significantly, in accordance with the views of Cotterall (1995) who described how autonomy can be encouraged through the gradual withdrawal of teacher support.”<br>“Overall, the learning journey for this “active learning” assessment leads to increased levels of motivation, control,		

				and confidence prior to the final assessment compared to baseline."		
Pope 2020[34]	Aim: To examine how specifications grading in an undergraduate dietetics course impacts students' learning and experiences.<br>Design: Cross-sectional.<br>Method: Qualitative, survey-based open ended essay type answers followed by focus groups.	USA	61 Dietetics students (survey narrative data) and 13 students in focus groups	"Traditional grading can be cumbersome for instructors, encouraging extrinsic motivation for students and hindering clear understanding of whether students have met course learning outcomes."<br>"Employing specifications grading may help dietetics educators foster self-regulation and mastery learning for students, as well as increase grading transparency."<br>'Specifications grading reinforced self-regulation and motivation, 2 key behaviors linked to improved student learning.'<br>"Specifications grading supported the planning phase of self-regulation because students needed to look at the syllabus and specifications to plot how they would achieve the grade they wanted on each assignment."<br>'I am the type of student that really enjoys everything organized and laid out right in front of me. Usually if things are not all laid out in front of me, I'll make my own checklist to make sure I have Everything done. Specifications grading helped me make sure all aspects of each assignment were completed before submission.'<br>'Students articulated the ways in which specification grading not only facilitated planning of how to complete the assigned task, it enabled them to assess their progress toward the overall goal. In other words, it fostered motivation, albeit extrinsic motivation.'<br>'Specifications grading invites students to hone intrinsic motivation because the schema has a built-in system of instant feedback through 1-level rubrics or specifications checklists that attempt to shift students' focus to mastery learning.'	Dietetics students	High<br>Rigor ++<br>Relevance ++

				‘I did not think I would like it at first because I usually get through classes putting less effort into smaller assignments and still getting A’s which I couldn’t do [with specifications]. What I found was that it actually made me care about the small assignments more and have a better connection to the class material.’<br>‘This suggests that courses with traditional grading structures that establish a hierarchy of value to assignments undermine student engagement and perhaps learning objectives of the course.’<br>‘I liked how it also made everything seem important in the way that they were all equal in number of points received. The reading responses were important to do, and I learned a lot from them, and they made me end up looking up for articles on similar topics.’<br>‘Specification grading is not a magic wand that will reorient students to learning for learning’s sake. However, an instructor’s use of specifications grading to measure achievement of learning outcomes may contribute to a paradigm shift in students’ motivational orientation, a shift from a grade orientation to a learning or mastery orientation.’<br>“Several students shared how specifications grading allowed them to be creative because they did not worry about the grade.”<br>“I dislike specifications grading very much as there is no real grade associated with completing an assignment. It’s either you did well, or you didn’t.” “No real grade” suggests that this student was still looking for a ranking compared with her peers and an A-F system. If she met the specifications and received a satisfactory grade, then she had mastered the assignment, but this still did not square up to her extrinsic motivation to receive a letter grade. Moreover, despite the		

				apparent benefits of specifications to a student's sense of engagement and learning, traditional grades continue to be seen as the ultimate motivator for learning.'<br>'I do like the fact that specification grading provides you with the information to get any desired grade, but I think this may cause students to become unmotivated to reach their full potential.'<br>'Even after this student listed the ways specifications was an improvement over traditional grading (transparency, stress reduction, intrinsic motivation, and student control), she still contended that for some students this might not be enough motivation.'<br>'Exposing students to more courses that use specifications grading may be 1 way to combat the ingrained socialization that traditional grades are more valuable or more motivating than content mastery.'<br>'Specifications grading is an assessment system that can help nutrition educators focus on students' achievement of course learning objectives and give each student more agency over learning and achievement.'<br>'If specifications grading is deployed more widely, it may shift perceptions of grading, motivation, and learning in higher education.'		
Sadowski 2020[48]	Aim: To explore the feasibility of adding video recordings of existing practical examinations to a physical diagnosis course and to evaluate students'	USA	50 Semestar-1 students who were enrolled in a 7-semester PA	'Students reported increased awareness of their own verbal and nonverbal communication strengths and weaknesses with the use of video-recorded practicals, as well as increased motivation to practice.'<br>Most students agreed with the statement "I am more motivated to practice my skills because of having seen my performance on a video" in the questionnaire.<br>'Physician assistant students expressed that viewing their own video performances pushed them to identify their mistakes sooner and motivated them to practice more.'	Physician Assistant Students	Low Rigor – Relevance +/-

	self-assessments and peer assessments of the activity.<br>Design: Cross-sectional.<br>Method: Quantitative, survey-based, exploratory study.		program participated			
Saunders 2019[44]	Aim: To measure student satisfaction with a collaboratively tested OSCE and student perceptions of strengths and weaknesses of the OSCE design.<br>Design: Cross-sectional, quantitative.<br>Method: Survey-based with free text comments.	Australia	332 Year-1 nursing students, 211 responses for qualitative data	'Collaborative Testing:<br>- Motivation - It helped to motivate us to study more, being with a partner it also encouraged us to do better.<br>- Responsibility for mark of peer - Having to study hard to ensure I didn't let the other student down.<br>- Feedback - Working together with another student that corrects me and reminds me of what I am missing. It is great to have the experience of teamwork & positive feedback.<br>- Improved performance - Practicing the skills in pairs great to get feedback as you were practicing you were able to get it right from the word go.<br>Clinical Relevance:<br>- Teamwork - Working as a team which replicates nursing environment. It replicates an actual clinical setting by working with another student=teamwork rather than learning the wrong thing.'<br>'Students also reported that working in pairs reduced their anxiety and increased their sense of responsibility and motivation, noting that it 'strengthened my self-confidence and 'encourages you to excel so you don't let your partner down.'	Nursing students	High Rigor + +<br>Relevance + +

				‘Collaborative testing motivates students to engage in peer learning as they prepare for the OSCE together, the interaction experienced through peer learning increases engagement and motivation’. (Mechanism)<br>‘Students in this study found peer work encouraging and in particular, were motivated by the responsibility to perform well for their partner. This motivation to perform well resulted in increased preparation with their partner, and is arguably where the real power of collaborative testing lies.’ (Mechanism)<br>‘The OSCE described in this paper was a high-stakes (40%) summative assessment. However, in the sense that students prepared collaboratively to close a performance gap through ongoing peer evaluation and provision of feedback, this OSCE design promoted principles of formative assessment’. (Mechanism?)		
Schüttpelz-Brauns 2020[39]	Aim: To identify institutional strategies related to serious test-taking behavior in low stakes assessment.<br>Design: Cross-sectional, quantitative.<br>Method: Survey with free text comments.	Germany	108,140 observations on test moments of medical students in a progress test and survey filled out by faculty.	‘The odds of taking the test seriously increases by 153% if there are consequences for not participating in the BPT as compared to no consequences for not participating. When the results of low test performance are discussed with the mentor, the odds of taking the BPT seriously are increased by 1423% as compared to a situation where the results of low test performance are not discussed with the mentor. In contrast, if students are given some choice about modalities of their participation (“Give choice”), their odds of taking the BPT seriously declines by about 1 - exp(- 4.25) = 99% as compared to not providing them with this<br>choice. However, this negative main effect is weakened by the variable “Presentation type”:<br>provided that students are given choice, the odds of taking the BPT seriously when the test was presented as an assessment as compared to being presented as an evaluation, is 11.21	Medical students and faculty	High Rigor + Relevance ++

				higher than the same odds for students who are not given choice.' 'Teachers play an important role in motivating students in low stakes assessment.' 'students are on different levels of motivation and therefore motivational strategies can have differentiated effects on different students. Curriculum planners do not only have to provide motivational strategies in medical education including low stakes assessments but also have to take strategies into account that address the different motivational levels from external regulation to intrinsic motivational level.' 'The strategies that were related to higher odds of taking the test seriously are (in decreasing order): discussion of low performance on BPT with the mentor, consequences for not participating, and give choices of place and date of test taking. Additionally, serious test-taking behavior occurred more if students were given choices and if the BPT was presented as assessment or if students were given no choices and if the BPT was presented as evaluation. Including discussing the results of low test performance with the mentor could work because talking to a faculty member about the low performance could show that performance is important to someone else and students might want to avoid talking about a lower performance. Depending on whether the student is in the introjection level or intrinsic level of the SDT model, her or his more serious test-taking behavior might be explained by avoiding the feeling of guilt (introjection level) or feeling more related (intrinsic level of motivation).' 'In contrast to these prior findings, in our study we showed that if students were given the choice of place and time for taking the BPT, they showed more non-serious test-taking behavior. This negative		

				relationship was lessened if the BPT was presented as assessment rather than as evaluation. The latter finding can be explained if students that showed more serious test-taking behavior in the combination of BPT as assessment at the same time as having no choice about the BPT were on the external regulation level of the SDT. If the BPT is part of the evaluation, students have to be on the intrinsic motivation level to show more serious test-taking behavior. Therefore, the components for the intrinsic motivation level are important here, as is freedom of choice.' 'We could show that strategies that can be assigned to different levels of motivation in SDT are related to more serious test-taking behavior in a low stakes assessment.'		
Sockalingam et al. 2017[36]	Aim: To explore the factors influencing LLL and motivation to learn during residency training and practice. Design: Cross-sectional. Method: Qualitative focus groups based (for residents) and interview-based (for faculty).	Canada	23 Psychiatry residents and 11 faculty/educators	"Respondents described a preponderance of extrinsic motivation factors during residency training, including exams and meeting program or supervisor expectations, and less of an emphasis on intrinsically focused factors impacting LLL, such as the enjoyment of learning and discovery." "They also commented that licensure exam in the more senior years was an external motivator and expressed frustration with its lack of clinical relevance." "Using data and information from external sources, such as peer feedback, was cited as a mechanism for increasing motivation for LLL." "Psychiatrists in all groups referred to external regulatory bodies and maintenance of certification programs as a motivating factor for ongoing LLL." Respondents also described their responsibility as physicians to deliver high-quality patient care as a key motivating factor in their LLL.	Psychiatry resident and faculty	High Rigor ++ Relevance +

Tey 2020[38]	Aim: To explore the educational impact of having the traditional long case as an educational assessment.<br>Design: Cross-sectional.<br>Method: Qualitative (focus groups for medical students and semi-structured interviews for examiners).	Australia	20 Final Year medical students and 6 examiners	'Engaging in the activity of a long case had an essential role in fostering students' clinical skills and served as a powerful driving force for them to spend time with patients.'<br>'Students had concerns about inter-case variability, but there was general consensus that the long case was valuable, with allocation of marks being an important motivator for students.'<br>'Exactly the skills that you need as an intern'.<br>'Teaching them the skills that they need to practise'.<br>'Students viewed the long case as an authentic task and contributed many specific examples of the ways in which long cases would assist them during internship, including at patient handover, during preadmission clinic, and when admitting new patients from the Emergency Department, which was described as just doing a mini-long case'.<br>'it feels real, cause you come up with an issues list, and management'.<br>'the long case was the only time I felt like I was actually learning to do medicine'.<br>'It's like all of medicine there's two types of study. There's study for the exams, and there's study for being a good doctor and a competent doctor. And the two don't necessarily seem tomatch up…I do think it falls into that how to be a good doctor', as opposed to 'how to do the exams well'.<br>'I'm glad that I've had the time to work on them in a safe kind of environment.'<br>'Examiners were universal in their opinion that the long case mirrored what doctors do in their everyday work'.<br>'Students described the long case as a "stretch goal" and "the pinnacle of what we were trying to do'.<br>'I've always told the students: the students that do the best are the students that talk to	Students and examiners	High<br>Rigor ++<br>Relevance ++

				the most patients, examine the most patients, see the most patients, spend the most time in the wards…patients teach you medicine, not textbooks. And I think the long case in some ways exemplifies that learning and that teaching.'<br>'I think because all of that variability exists, it's a really good assessment in making us do it, but it would seem unfair if it was – it had a large weight.'<br>'…I found the whole thing really stressful but I also think it's probably our best assessment, even though I hated it a lot'.<br>'The meaning ascribed to the long case score by students and examiners was an important concept which became apparent across the focus groups and interviews. There was general agreement that long cases had to be graded for students to "take it seriously" [Examiner 6]. Some students volunteered that if long cases were formative rather than summative, they would have "put much less effort in" [Student 3] and there would have been "decreased motivation" [Student 18]. Although a small number of students wished for long cases to be purely formative as it was "such a rich learning experience" [Student 7] in itself, they had great insight into the fact that their more results-focused peers would be far less motivated to tackle the long case if it did not count towards their final marks'.<br>'Some people would fully rise to the occasion, because at the end of the day you just want to be a good doctor and you want to impress the doctors that you work for. But other people would fall so far short of jumping that hurdle and would just be like, oh, I don't care about that, I've got to get whatever they mark OSCEs out of, 40 out of 40 for my hypertension OSCE dance [laughter from group].'<br>'Long case assessments had the important effect of motivating our student informants to spend time with patients.'		

				'Although long cases acted as a powerful motivator for students to see patients on the wards, the unintended "flip side" was that a small number of students appeared to deprioritizeopportunities for brief patient interactions which fell short of a full-length long case.'		
Vinall 2020[30]	Aim: To determine whether inclusion of individual end-of-class formative quizzes could encourage students to reflect on and address deficiencies and improve academic performance. Design: Cross-sectional, quantitative, single-blinded, randomized, controlled, crossover study. Method: Survey-based.	USA	97 Year-1 Pharmacy students	'Quizzes can improve summative exam performance for below average first year pharmacy students, and improve self-reflection and student motivation to study.' 'The inclusion of quizzes improved students' motivation to study; 83% of students strongly agreed or agreed that poor performance on quizzes would make them feel compelled to study more (survey question 5, Table 1), 61% of students strongly agreed or agreed that poor performance on quizzes would make them feel compelled to attend review session (survey question 6, Table 1), and 28% of students strongly agreed or agreed that poor performance on quizzes would make them feel compelled to attend office hours'. 'Our survey data indicate that the including individual formative end-of-class quizzes comprising of higher level Bloom's questions promoted student self-reflection and motivated students to implement self-directed corrective actions, and it is likely that the increase in performance of lower-performing students that we observed was at least in part due to these individual formative quiz-induced behaviors.'	Pharmacy Students	Low Rigor + Relevance +/-
Weller 2014[28]	Aim: To describe the meaning of examination preparation as experienced by individual trainees.	Australia and New Zealand	20 Anesthesia specialization trainees	"Examinations provided a framework for learning and were a key motivator to ensure that the required level of knowledge was gained. Some also acknowledged that it "forced" them to spend time on learning that they otherwise may not have prioritized." "Motivation: The exams are very powerful drivers for knowledge accrual and the oral exams are very powerful	Anesthesia trainees	High Rigor ++ Relevance ++

	Design: Cross-sectional, qualitative.<br>Method: Interview-based.			drivers for learning to verbalize your thought processes, which is important for crisis management which is a major part of our job.” “I think they’ve forced me to learn things that, being naturally lazy, I wouldn’t have myself.” “The exams give you an external structure…. It also has a deadline, which gives you motivation.”<br>“There was an element of endurance associated with the Primary Examinations (PEX), in that it was something that had to be done (a “hurdle”) that was not particularly enjoyable.”<br>“The Final Examinations (FEX) was recognized for its strong clinical focus, with interviewees reporting that it assessed their clinical expertise and played a key role in ensuring that the<br>program produced safe and competent anesthetists.”<br>“While several found it more enjoyable, some found the less explicit and directed curriculum<br>for the FEX challenging.”<br>“Anesthesia examinations are an important extrinsic motivator for trainees to acquire knowledge required for specialist practice, without which some may never study the curriculum in depth.”<br>“One issue raised in our study was the potential to use approaches aimed just at passing the exam rather than at meaningful understanding of the curriculum at least for some of their study time.”<br>“Choosing what to learn was based on past exam questions and gaming about likely exam questions, rather than what was essential knowledge for clinical practice.”<br>“The ‘cram and dump’ approach associated with some aspects of specialist examinations may not be a very effective or indeed humane approach to promote acquisition of the		

				necessary knowledge, skills and attitudes required for specialist practice.”		
Weurlander 2012[27]	Aim: To provide a greater insight into how formative assessments are experienced and understood by students and compare how students perceive the effects of individual vs. group assessment.<br>Design: Cross-sectional, qualitative.<br>Method: Written accounts and group interviews.	Sweden	17 Year-2 medical students	‘Our findings suggest that formative assessments motivate students to study, make them aware of what they have learned and where they need to study more.’<br>‘Our findings show that formative assessments are an important tool for students’ learning in three areas: motivation to study, awareness of their own learning and the effects on learning, in terms of both processes and outcomes.’<br>‘Formative assessment influenced the students’ motivation to study in several ways. For many students, the formative assessments seemed to act as external motivators. Students felt pressure to study for the assessment and said that they needed some stress or deadline to motivate them to study, especially early in a long course. I need the extra stress to go back because even if I have read it before I need this. . .‘assessment stress’ to go back and rehearse once or twice more to make it stick (individual assessment, group interview, ind. assm, interview). This suggests that the assessment triggers extrinsic aspects of motivation which, in turn, have an impact on certain actions, in this case to study and practice for the assessment.’<br>‘There was also evidence of intrinsic motivation such as a growing interest in the subject as a result of studying for the assessment. Students noted that they retained information more easily when they were interested in a subject. Moreover, an assessment task can be stimulating and challenging in itself, and thereby trigger intrinsic motivation, as was the case with the group assessment. The group assessment was fun and challenging, and gave the much longed-for overall picture that is so difficult to put together on your own (group assm, wr reflection).’	Medical students	High<br>Rigor ++<br>Relevance ++

				‘the individual assessment is that it was primarily a trigger for extrinsic motivation.’<br>‘A reward in the form of a few extra credits in the final exam seemed to motivate some students to study for the individual assessment. Students also seemed to act strategically, in that they spent time studying rather than doing other things and focused their studies on the content of the lectures rather than following the syllabus.’<br>Motivation to study - External pressure and reward trigger extrinsic motivation in individual assessment. Fear of disclosing lack of knowledge triggers extrinsic motivation and the task (group assessment) itself triggers intrinsic motivation.<br>‘Group assessment are two things: firstly the assessment format itself seemed to stimulate intrinsic motivation and secondly, it helped students to connect their knowledge to real-life contexts and it contributed to a sense of ‘wholeness’.<br>‘Many students described the assessment task as fun, challenging and interesting. However, students also seemed to feel the pressure to study for this assessment, which indicates that assessment tasks can trigger both extrinsic and intrinsic motivation.’ (Mechanism)<br>‘the individual assessment in the present study appeared to be an important factor in the students’ motivation to study. This could be due to the fact that the individual assessment came only two weeks into the course and the students had recently studied hard for the final exam of the previous course.’ (Context/Mechanism)<br>‘The group assessment may also have acted as a tool for learning because of the design of the task itself, which captured the students’ interest and focused on problem-solving and practical application.’ (Mechanism)<br>‘The group assessment appeared to capture the students’ interest; they found the task challenging and relevant for their		

				future profession. Interest has, in turn, been found to be closely linked to intrinsic motivation which is often associated with a deep approach to<br>Learning.'<br>'The strong focus on factual knowledge in the individual form of assessment and the extrinsic motivation felt by students may steer them instead towards a surface approach to learning.<br>The data, however, indicates a movement from extrinsic to intrinsic motivation. Even though students felt pressured to study for the assessments, they became interested in the subject as they learned more. Moreover, the findings suggest that students have the intention to understand but the pathway to reach understanding involves memorization, which the individual assessment encouraged.' (Mechanism)<br>'The findings of this study support the idea that formative assessment methods can act as tools for learning by affecting students' motivation to study and by making them aware of their own learning, thus contributing to their learning process.'		
Yoneda 2020[47]	Aim: To describe an innovative assessment method, the error-detection examination, and students' evaluation of the process.<br>Design: Cross-sectional, Quantitative.<br>Method: Survey-based.	Japan	85 Year-5 Dental students	'The questionnaire revealed that this method of testing applied knowledge was new to students and helped them to identify knowledge gaps.'<br>'About 81% said the test was useful to show them their level of knowledge. After the error detection examination, 80% of students also reported that they wanted to study conservative dentistry thoroughly again'.<br>'They also said that this form of active learning had increased their motivation to study<br>conservative dentistry'.	Dental students	Low Rigor +<br>Relevance +/-

Supplemental Digital Appendix 3

Context-Mechanism-Outcome Data Extracted From the Included Studies

Context	Mechanism	Outcome
Stimulating autonomous motivation		
Assessment was fun and challenging<br>- because the assessment task was fun, interesting, challenging.[26,27,36,40,45]<br><br>For some students the motivation to learn comes from their personal gain and achievement or 'love of learning' or own satisfaction or a passion for the topic.[26,40]	The assessment was fun and challenging and triggered intrinsic motivation by itself and because of the group component also provided a holistic picture which would be difficult for a student to do on his own.[27]<br><br>Some students would study for assessments even if they were not graded as they just want to be good doctors.[38]<br><br>Because the students could choose the topic that they were personally interested in, they could weave their interests in.[45] The students were more driven by personal motivation and changed their learning approach.[45]<br><br>Students worked to a schedule because of their intrinsic motivation.[32]	Intrinsic motivation surfaced after the assessment task was over.[26]<br><br>The students were more driven by personal motivation and changed their learning approach.[45]
Seamless introduction of assessments<br>- If assessment is seamlessly introduced without interrupting students' learning process.[46]		
Multiple and longitudinal assessments<br>Having innovative mastery-based assessments for multiple courses[34] and longitudinally over multiple measurement points.[33]	Formative assessments motivate students by making them aware of what they know and what to study more.[27]	Effort<br>- Less effort on formative assessments as compared to summative assessments.[38]

Student Autonomy in<br>- determining the grade they would like to earn from a set of parameters[34]<br>- pass/fail grading only[34]<br>- instructor-determined mastery criteria[34]<br>- meeting these criteria is required to obtain a passing grade[34]<br>- provision of multiple attempts to pass[26]<br>- choice of assignments[34]<br>- in learning their topics of interest[40,45]<br>- place and time of assessment[39]	Specifications grading stimulates autonomous motivation by providing instant feedback through rubrics and shifts the focus to mastery learning.[34]<br><br>Specifications grading fosters self-regulation and motivation, which stimulate student learning and helps students understand where they stand vis a vis the goal.[34]<br><br>Autonomy in learning<br>- Use of portfolio made students approach learning through a more personal motivation and not rely only on lecture material for their study.[45]<br><br>- Choice of topics to study/add in the portfolio removed boundaries (to be confined to the curriculum), allowed for personal exploration and made students appreciate the basic sciences even more.[45]<br><br>- As students progressed through the planning process and became more independent, their motivation levels increased.[45]<br><br>- Autonomy in learning made students independently develop their own skills and made them more motivated.[40]<br><br>Seeing their performance on video recording motivates students to practice their skills.[48]<br><br>Kahoot! Sessions motivate students to study more, focus on important concepts, and reflect on their learning.[31]	Creativity<br>- Assessments (with specifications grading) stimulated creativity when students did not worry about the grade[34]<br><br>Effort<br>- Specifications grading improved effort on completing work in a timely fashion and made the students care about the smaller assignments and connect better with the class material.[34]<br>- Error detection assessments encourage students to study harder.[47]<br><br>Specifications grading fosters self-regulation and mastery learning.[34]<br><br>Formative assessments foster self-regulation and time management skills[41] and self-reflection and self-directed corrective actions.[30]<br><br>Portfolio fostered a better learning approach through fostering personal motivation.[45]<br><br>Choice of topics to study/add in the portfolio made students appreciate the basic sciences even more.[45]

Building competence<br>- [33,44]Assessment of clinical expertise[28]<br>- Reciprocal peer coaching[41]<br>- Timely, External and multiple source feedback[31,49]<br>- Active learning assessments[40,47]<br>- Collaborative testing[44]	Reciprocal peer coaching has the capacity to increase motivation by giving immediate feedback.[41]<br><br>Students appreciated the face to face feedback they received in workplace based assessment. This improved their competence and confidence.[26]<br><br>Getting feedback from patients and colleagues motivates students.[49]<br><br>Getting to know the correct answer immediately after the assessment, like in Kahoot!, stimulates students to focus more on the questions and stimulates their motivation.[31]<br><br>Multiple Choice Questions followed by explanatory feedback enhanced the learning process and sustained student motivation.[46]<br><br>Active learning assessments led to increase in the skills and confidence and increased motivation after each stage in the learning journey was completed.[40,47]<br>Active learning assessments engaged students not only supported knowledge building but also engaged them cognitively and emotionally.[42,45] They generated enquiry by providing the students the opportunity to formulate questions.[45]<br><br>Error detection mechanism helped students to identify their knowledge gaps.[47]<br>Collaborative testing helped in closing a performance gap through constant peer evaluation and feedback.[44]	Feedback leads to a feeling of competence, increased confidence and improvement in performance.[26]<br><br>Explanatory feedback following an assessment enhances learning process and motivation.[46]<br><br>Formative assessments improve performance of low performing students.[30]<br><br>Active learning assessments made students aware of their developing skill set.[40]<br><br>Assessments inspired an interest in the subject.[27]
Setting goals<br>– Either shared goal[41] or individual goal[40]		Entrustment scales can stimulate students' goal setting and learning.[33]

Reflection on assessments<br>- Stimulating learners to reflect over assessments[33]	Reflection on assessments provides a better picture on the students' development and improves their motivation, learning and well-being.[33]	Reflection on assessments provides a better picture on the students' development and improves their motivation, learning and well-being.[33]
Authenticity with practice[26,38]<br>Practice-related drive[28,35,37,38,49]	Authenticity with practice<br>- Students found the long case to be authentic and the hallmark of the actual practice. This motivated them intrinsically.[38]<br>- Choice in portfolio allowed students to include their personal experiences into their medical study and significantly motivated them.[45]<br>Portfolio assessment was perceived as an authentic task related to future practice and hence developed skills and professionalism.[45]<br><br>Authentic tasks related to future practice were motivating as they provided a sense of wholeness, fostered clinical skills, captured students' interest and encouraged team work.[27,38,41,44,45]<br><br>Professional responsibility as physicians motivated students for lifelong learning.[36]	Authenticity with practice<br>- Created search for deeper understanding[26], Provided significant motivation for learning.[26]<br><br>Video recorded performance assessments led to better error identification and more effort to practice skills.[48]<br>Long case assessment motivated students to talk to and spend more time with patients.[38]<br><br>Portfolio helped in developing skills and professionalism.[45]
Sharing and shared/group learning and responsibility<br>- Shared Learning[39]<br>- Shared goals[41]<br>- Group work and responsibility[27,41,44]<br>Co-constructed assessments<br>- When (sustainable) assessment is jointly constructed between learners and teachers[45]	Knowing and understanding what was expected in the assessment helped students to improve their work on assignments.[42]<br><br>Peer learning helped students to identify gaps in their knowledge, created constructive friction and moved them into Vygotsky's Zone of Proximal Development, which enhance their learning.[41]<br><br>Peer assessment functioned as an extra motivational strategy for individual students to contribute to the group in a meaningful way and provided experience of teamwork.[29,44] The peer assessment	Peer learning made students more knowledgeable about the assessment and encouraged them to improve their work more.[42]<br><br>Peer assessments makes students read and understand material better, think more, read and reflect more.[43]

	process empowered students, motivated them, and increased their confidence and engagement in learning.[42]<br><br>Peer learning and preparing for assessment together.[44]<br><br>Engagement in peer learning leads to interaction which leads to engagement & motivation (collaborative testing[44])<br><br>Group assessment helped students to put a helicopter view of the topic together, which would have been hard to achieve on their own.[27]<br><br>Learning in a group with a shared interdependent goal made students feel personal responsibility for the group work and bring more knowledge and effort in their contribution.[41]<br><br>Failure leads students to study harder.[33]<br><br>Not passing their entrustment assessment made students feel frustrated.[33]	Peer learning and interaction increases student motivation and engagement and skill development.[44]<br><br>Increased engagement and motivation due to collaborative testing.[44]<br><br>Group work promoted higher quality of work and attendance[29], and engagement and motivation[44].
Individual variation in<br>- Effects of different types of assessments on the participants[26]<br>- Baseline motivational levels of students[39]	Individual variation<br>- Some students perceived generating ideas as a competitive process, while others perceived it as an opportunity to reflect on their ideas and making comparisons.[40]	
Value of assessment – holds certain personal value[27,31,45]		
Stimulating controlled motivation		
External motivators –<br>- The presence of a system for assessments is an external	Assessments trigger controlled motivation which further makes students study and practice for assessments.[27,]	The existence of examinations force students to spend time on learning, including material that is important

motivator for study or learning or preparing for the assessments. These can be formative[31,32]or summative[26,37] assessments[36].[27,28] .<br>- Presence of grades[34,38], Strong focus on factual knowledge[27,]<br>- Competitive element[26,31]<br>- Fear of disclosing lack of knowledge[27] or failure[26], Pressure – Performance pressure[27], time pressure[26,27,28,35]and emotional pressure[27], External pressure and/or reward[27], the desire to fulfill supervisors' expectations[36]<br>- When consequences are coupled with assessments[39]<br>High stakes assessment[26], Licensure exams[36]	External pressure and reward function as triggers for controlled motivation.[27,29,31,38,41,42] They also lead to anxiety and frustration.[26,27,40,41]<br><br>The existence of examinations force students to spend time on learning, including material that is important for practice[28], and also study and practice with a goal to pass assessments[27,].<br><br>Focus on factual knowledge in assessments and controlled motivation leads to surface learning.[27,31]<br><br>Grading of assessments motivates students to submit high quality work.[43]<br><br>If an assessment question is asked in Kahoot!, students perceive it to be important for final examinations, and study it harder.[31]<br><br>Peer assessment led students to compare their performance with their peers[42] and fear receiving low marks if they did not contribute[29] and motivated them to learn.<br><br>Students study harder for the reward of extra credits[27] and the fear of getting lower grades or failure[26,29] and poor performance[30].<br><br>Wanting to know what their peers thought of their work, prompted them to reread their work in light of the feedback from their peers.[42]<br>Competition motivates students and makes them prepare for assessments.[26,31]<br>Focus on entrustment as the assessment outcome may enhance learner perceptions of being judged continuously and of assessments may be perceived as being summative rather than	for practice[28], and also study and practice with a goal to pass assessments[27,31].<br><br>Controlled motivation for assessments leads to surface learning.[27]<br><br>Students feel the pressure to study for assessments.[27] They also feel anxiety, frustration and stress.[26,27,33]<br><br>Peer assessment led students to compare their performance with their peers and motivated them to learn.[42]<br><br>Competition makes students prepare for assessments.[31]<br><br>Students study harder for the reward of extra credits[27] and the fear of getting lower grades or failure[26,29]and poor performance[30]<br><br>Traditional as well as specifications grading increase students' controlled motivation.[34]<br><br>Formative assessments stimulate students' controlled motivation and achievement.[31]

	formative. Thus can undermine the learning potential of an assessment programme based on trust and psychological safety.[33]<br><br>Students who learn by rote memorization without understanding and regurgitate the material during the exams tend to fare better on MCQs.[26]<br><br>Formative assessments stimulate students' controlled motivation and hence achievement.[31]<br><br>Poor performance compelled students to study more.[30]<br><br>Because of the focus of entrustment language on autonomy and unsupervised practice, it can lead to a 'performance culture' at the expense of learning and development.[33]<br><br>Students studied hard in collaborative testing as they did not want to disappoint their partner students. This also strengthened their confidence and made them excel.[44]<br><br>The existence of external regulating bodies and regulations stimulated lifelong learning.[36]<br><br>Examinations provided a framework and ensured, even forced the students to acquire and prioritize the required knowledge.[28]<br><br>Even though students felt pressurized to study more by assessments, they started liking the subject along the way.[27]<br><br>Students are inclined to pick easier Direct Observation Procedural Skills in order to do better on assessments.[26]	Entrustment scales carries the risk of being turned into a tick box exercise, i.e. strategic behavior, by students.[33]<br><br>If entrustment language is focused on autonomy and unsupervised practice it can lead to a 'performance culture' at the expense of learning and development.[33]<br><br>Focus on entrustment as an outcome of assessment can undermine the learning potential of an assessment programme.[33]<br><br>Assessments drive strategic learning from the students to pass exams.[26,27,28,32,33]<br><br>Failure in assessments drives away inspiration and motivation.[35]<br><br>Failure on entrustment scale reduced students' sense of self-efficacy and colored their reflections of workplace based learning.[33]<br><br>High stakes assessment drives the wrong kind of motivation[26]<br><br>Undermining of student engagement and learning objectives of a topic

	Specifications grading supports self-regulation in the planning phase and helps students study to achieve the grade that they want.[34]<br><br>Choosing what to study was based on previous exam questions rather than on what knowledge is required for practice.[28]<br><br>MCQs encouraged surface learning approach owing to the high stakes attached to them, while Direct Observation Procedural Skills encouraged a deep learning approach as the stakes attached to these were lower.[26]<br><br>When traditional grading is employed for assessments, students value the assessment in the hierarchy of the weightage this assessment carries in the total picture. This influences their engagement with the topic and also how much importance they give to the learning objectives.[34]<br><br>Making EPA based assessments high stakes may give rise to strategic behavior of students.[33]	happens in relation with the importance and weightage the topic carries in the assessment system.[34]
Timing of assessment<br>- Motivation dipped towards the end of the assessment[40]<br>- Timed at two weeks after the course started when students had recently studied hard for the final exam of the previous course.[27]	Timing of the assessment influences on how students put effort in it.[27]	
Conflicting agendas<br>- conflict between preparing for their clinical profession	Individual variation<br>- Some students perceived generating ideas as a competitive process, while others perceived it as an opportunity to reflect on their ideas and making comparisons.[40]	Conflicting agendas made students abandon what they found interesting to study and focus on the assessment.[26]

and preparing to pass final examinations[26,38].		

Supplemental Digital Appendix 4

Specific Assessments Used in the Included Studies

Reference	Type of assessment used and its description
Ahmad 2018[32]	No innovative form of assessment
Alkhamees 2020[37]	No innovative form of assessment
Asghar 2010[41]	**"Reciprocal Peer Coaching** is a form of co-operative or peer-assisted learning that encourages individual students in small groups to coach each other in turn so that the outcome of the process is a more rounded understanding and a more skillful execution of the task in hand than if the student was learning in isolation."
Bengtsson 2010[35]	No innovative form of assessment
Casey et al. 2011[42]	**Peer Assessment** – "The process involved second year Bachelor of Nursing Science students developing marking criteria and marking two of their colleagues' assignments anonymously."
Cobb et al. 2013[26]	DOPS – **Direct Observation Procedural Skill**, and MCQ – Multiple Choice Questions. "DOPS are used as a form of workplace-based assessments to examine the performance of practical and clinical skills of final year students."
Elliott 2005[29]	**Self and Peer Assessment** – "Self-assessment is defined as students assessing their own work and peer-assessment as students assessing the work of others within a group."
González-Gil 2020[49]	**360-degree evaluation** – "The main difference between a 360-degree evaluation and more "traditional" evaluation methods lies in the fact that it considers different perspectives and sources of information which, when inter-related as a network, makes it possible to obtain a comprehensive and integrated view of the process, the organization, or the professional under evaluation."
Harris 2011[43]	**Peer Assessment** – "Peer Assessment has been defined (33) as an arrangement for peers to consider the level, value, worth, quality, or successfulness of the products or outcomes of learning of others of similar status."
Ismail 2019[31]	**Kahoot! as formative assessment** – "Kahoot!, a real-time platform for game-based learning, is a free formative assessment tool that has been widely used in education."
Leadbeatter 2018[45]	"The portfolio of three assessments designed using the principles of **integrative learning, scaffolded learning, and sustainable assessment** was introduced in the Bachelor of Oral Health Therapy program."<br>- "Integrative learning is an approach to learning based on the idea that learning is not limited to a specific setting, such as an academic class or course."<br>- "Scaffolded learning is another approach that can help students move away from dependent relationships with course materials and teachers. Throughout a scaffolded task, responsibility is transferred to students, and learning becomes increasingly integrated as fading (or taking down) of the scaffold occurs."

	- "Sustainable assessment is an approach to assessment that positions students as active learners who need lifelong learning skills. Rather than a one-sided act done to students, assessment is acknowledged as being jointly constructed between learners and teachers."
Makransky 2020[46]	"A desktop virtual reality (VR) laboratory simulation on the topic of genetics, with **integrated assessment** using multiple choice questions based on item response theory (IRT) and feedback based on the cognitive theory of multimedia learning."
Martin 2020[33]	**EPA-based assessments** – "Entrustable professional activities (EPAs)are activities or tasks that operationally define a profession and require the integration of numerous clinical competencies. Therefore, competence assessment in clinical settings around the world is increasingly EPA-based, defining entrustment as an assessment outcome."
Paxman 2011[40]	"**Active learning" assessment** – "This active learning assessment used a 5-staged learning journey model and involved students in planning and preparing for, then hosting a stall on a self-determined topic relevant to food, health or nutrition. The assessment consisted of 5 main stages: a briefing, thought shower, oral business proposal presentation, a feedback stage, and Nutrition Fair.
Pope 2020[34]	'**Specification grading** is defined with the following Contract Grading (CG) elements: (1) students determine the grade they would like to earn from a set of instructor-determined parameters, (2) assignments are graded pass or fail, (3) mastery criteria are determined by the instructor, (4) meeting these criteria is required to obtain a passing grade, (5) students are provided multiple attempts to earn a passing grade for assignments, and (6) students choose from a variety of assignments.'
Sadowski 2020[48]	**Self-assessment and peer assessment**
Saunders 2019[44]	**Collaborative testing** - A collaborative testing approach, where students are examined together and share the subsequent result. Collaborative testing involves the completion of a test or exam by two or more students, which is assessed by educators. As per collaborative testing design, students could discuss the skill during the OSCE and offer each other<br>verbal support without penalty. Both students received the same mark.
Schüttpelz-Brauns 2020[39]	**Low stakes Progress Test**
Sockalingam et al. 2017[36]	No innovative form of assessment
Tey 2020[38]	**The traditional long case**, used to evaluate medical students for over 150 years [2], is a form of clinical assessment which<br>requires a student to spend approximately 1 h with a patient, unobserved, then to present a summary of their history and examination findings to examiners who ask them questions about the case.

Vinall 2020[30]	**End-of-Class Formative Quizzes**
Weller 2014[28]	No innovative form of assessment
Weurlander 2012[27]	**Formative assessment** is defined as activities where judgements are made about the quality of students' achievements, and where this information is used to facilitate student learning.
Yoneda 2020[47]	**Error detection examinations** – The students were given error detection sheets. These sheets presented detailed written treatment procedures for typical cases, but some steps were purposefully written incorrectly. Students had to identify and amend these incorrect steps in the procedures. After correcting them, each student gave a presentation on their corrections to the group. This was followed by delivery of a summary of the ideal answers and a short lecture by a teacher.
